# Supplementary material for: What are young Indians saying about mental health? A content analysis of blogs on the It’s Ok To Talk website
Source: BMJ Open. 2019 Jun 22;9(6):e028244. doi: 10.1136/bmjopen-2018-028244 (PMC6596971; doi:10.1136/bmjopen-2018-028244)
Supplement: Author's manuscript [file bmjopen-2018-028244.draft_revisions.pdf]

BMJ Open is committed to open peer review. As part of this commitment we make the peer review history of every article we publish publicly available.

When an article is published we post the peer reviewers' comments and the authors' responses online. We also post the versions of the paper that were used during peer review. These are the versions that the peer review comments apply to.

The versions of the paper that follow are the versions that were submitted during the peer review process. They are not the versions of record or the final published versions. They should not be cited or distributed as the published version of this manuscript.

BMJ Open is an open access journal and the full, final, typeset and author-corrected version of record of the manuscript is available on our site with no access controls, subscription charges or pay-per-view fees (http://bmjopen.bmj.com).

If you have any questions on BMJ Open's open peer review process please email info.bmjopen@bmj.com

BMJ Open

What are young Indians saying about mental health? An analysis of blogs on the It's Ok To Talk website

Journal:	*BMJ Open*
Manuscript ID	bmjopen-2018-028244
Article Type:	Research
Date Submitted by the Author:	19-Dec-2018

Complete List of Authors:	Gonsalves, Pattie; Sangath,<br>Hodgson , Eleanor<br>Pal, Sweta<br>Sharma, Rhea<br>Naslund, John; Harvard Medical School, Department of Global Health and Social Medicine<br>Michelson, Daniel ; University of Sussex, School of Psychology<br>Patel, Vikram; Harvard Medical School, Department of Global Health and Social Medicine

Keywords:	MENTAL HEALTH, QUALITATIVE RESEARCH, Child & adolescent psychiatry < PSYCHIATRY

SCHOLARONE™
Manuscripts

Title of the article
What are young Indians saying about mental health? An analysis of blogs on the It's Ok To Talk website

Full name, postal address, e-mail and telephone number of the corresponding author:
Pattie P. Gonsalves
Sangath, C-1/52, First Floor, SDA, New Delhi, India
pattie.gonsalves@sangath.in
91.9899419704

Full name, department, institution, city and country of all co-authors:

	Full Name	*Department*	*Institution*	*City*	*Country*
1	Pattie Pramila Gonsalves	-	Sangath	New Delhi	India
2	Dr Eleanor Sara Hodgson	-	Sangath	New Delhi	India
3	Sweta Pal	-	Sangath	New Delhi	India
4	Rhea Sharma	-	Sangath	New Delhi	India
5	Dr John Naslund	Department of Global Health and Social Medicine	Harvard Medical School	Boston	USA
6	Dr Daniel Michelson	School of Psychology	University of Sussex	Brighton	UK
7	Dr Vikram Patel	Department of Global Health and Social Medicine	Harvard Medical School	Boston	USA

Word count, excluding title page, abstract, references, figures and tables: 4937

Keywords: Young people; mental health; social media; qualitative research

For peer review only

ABSTRACT

Objectives: This study explores young Indians' submissions to a youth mental health website, www.itsoktotalk.in through a thematic content analysis.

Setting: The study included submissions made to the It's OK to Talk web platform during the first year of its operation (April 2017 - March 2018).

Participants: 37 submissions by 33 authors aged 19-31 years (mean age 22 years) from 7 Indian cities (New Delhi, Lucknow, Bengaluru, Mumbai, Pune, Hyderabad and Haryana) were included for analysis. Eligible submissions were English-language first-person accounts of self-identified mental health problems, submitted in any media format for online publication by authors aged 18-31 years and of Indian nationality. We also collaborated with a focus group that comprised of eight study participants who were invited to participate in the coding process and preparation of final manuscript.

Results: Four thematic categories were identified:1) Living through difficulties; 2) Mental health in context; 3) Managing one's mental health; and 4) Breaking stigma and sharing hope. Overall, the participants expressed significant feelings of distress and hopelessness as a result of their mental health problems; many described the context of their difficulties as resulting from personal histories or wider societal factors, a general lack of understanding about mental health and prevalence of negative or stigmatising attitudes. Most participants expressed a desire to break down stigma.

Conclusions: It is possible that the process of communicating the experience of mental health and distress through blogging and other media may be a way of assisting young people with mental health problems to feel heard and connected with others. Personal narratives offer a window into young people's priorities and challenges in their own words and offer nuanced key messages for awareness and to encourage help-seeking for future anti-stigma public engagement efforts, both online and through on-ground efforts.

ARTICLE SUMMARY

Strengths and limitations of the study

- Strengths
  - qualitative analysis of first person accounts can illuminate numerous issues that are unlikely to arise in a clinical encounter
  - collaborative study design involving youth participants in the research, coding process and preparation of manuscript
- Limitations
  - a convenience sample resulting in a narrow demographic cohort of self-selected, highly educated, English speakers
  - mental health problems written about were self-reported and could not be differentiated into diagnostic or other clinical categories

For peer review only - http://bmjopen.bmj.com/site/about/guidelines.xhtml

SUMMARY BOX

What is already known?

- India has the world's largest population of young people ages 10-24, and mental health problems are the leading health concern for this group
- Recent attention has focused on social media as a vehicle for mental health promotion and stigma reduction particularly among young people
- Interest is building on the ways in which online social networks may be harnessed to support recovery from mental problems

What are the new findings?

- Four thematic categories emerged:1) Living through difficulties; 2) Mental health in context; 3) Managing one's mental health; and 4) Breaking stigma and sharing hope
- Most participants expressed a personal desire to break down stigma

What do the new findings imply?

- Communicating the experience of mental health and distress through blogging and other media may be a way of assisting young people with mental health problems to feel heard and connected with others
- Personal narratives offer a window into young people's priorities and challenges and offer nuanced key messages for awareness and to encourage help-seeking

For peer review only

INTRODUCTION

"I hope that reading my story might help others feel less alone. It is important to know that other people are fighting the same battles. This is for anyone who feels stuck, overwhelmed or hopeless. You are not alone, just try to hold on." - (Author, female, 24, www.itsoktotalk.in)

India has the world's largest population of young people ages 10-24**[1]**, and mental health problems are the leading health concern for this group. The negative impacts of youth mental problems are starkly reflected in the strong association between poor adolescent mental health and long-term social disability, while suicide is the leading cause of death for 15-24 year olds in India**[2-4]**. The sheer scale of the youth population in India (in excess of 350 million) presents unprecedented opportunities for health, economic and societal gains through primary and secondary prevention efforts centred on youth mental problems. Recent attention has focused on social media as a vehicle for mental health promotion and stigma reduction at a population level, particularly among young people. This reflects the developmental significance of peer influence in the adolescent years and the rapid uptake of digital technology by young people across the globe**[1, 5]**. Interest has also concentrated on the ways in which online social networks may be harnessed to support recovery from mental problems**[6]**. Such opportunities are especially relevant in India where rapidly increasing numbers of young people are becoming connected to technology and social media despite varied access**[7]**. At the same time, fewer than 10% of young Indians have access to formal mental health services, highlighting an urgent need to identify innovative strategies to promote mental health for this age group nationally and in low- and middle-income countries more generally**[8-9]**.

Sharing mental health stories online

*"Stories are essential as a means of how scientific knowledge, in its generality, can be applied to individuals in their particularity."***[10]**

"Social media" refers to websites and applications that enable users to create and share content or participate in social networking**[11]**. It is inexpensive and relatively easy to use, enabling individuals to bring personal experiences into the public domain and thereby impact attitudes among larger groups**[12-13]**. Betton et. al. (2015) argue that in fact it is individuals, rather than institutions, who are leading the way in bringing mental health conversations into online spaces. Individuals with stigmatised illnesses often avoid seeking healthcare**[14]** and reading or hearing about other people's experiences can challenge stigma**[15]** and affect health-related decision making and adjustment to an illness**[5]**. Young people with mental health difficulties report that one of the primary reasons for connecting with others online is to feel less alone**[16-18]**. Research also shows that young people may be more inclined to seek help for their mental health if they feel able to express their feelings, have some knowledge about mental health issues and available sources of support**[19]**.

About "It's Ok To Talk"

The It's Ok To Talk website (www.itsoktotalk.in)**[20]** was launched on WHO World Health Day (7th April) 2017 as part of a public engagement initiative by the Indian NGO, Sangath. It took inspiration from the theme for that year, "Let's Talk", emphasizing the central role of disclosure "as a vital component of recovery" from depression**[21]**. Following from evidence that disclosing a mental health problem to others can be an important approach to addressing stigma**[22-23]**, the platform was designed in consultation with young people aged 15-24 in New Delhi as a web-based social media space for young people to share their stories of mental health and raise awareness about youth mental health problems. Authors were given the choice to submit any kind of media and publish anonymously or to include their name, location and gender.

For peer review only - http://bmjopen.bmj.com/site/about/guidelines.xhtml

About this study

Existing research on youth mental health has focused largely on understanding the barriers faced in accessing care and experiences of services, while comparatively few studies have explored first person accounts of the lived experience of mental health difficulties**[24]**. Qualitative analyses of first person accounts can illuminate numerous issues that are unlikely to arise in a clinical encounter**[25]** and may offer potential to inform anti-stigma messages and guide further social media platform development. This paper employed a qualitative content analysis to examine the submissions of young Indians with mental health problems to www.itoktotalk.in. An important feature of this study is the collaboration with participants through the coding process and preparation of final manuscript to ensure the analysis stayed as true to their narratives as possible. The goal of the study is to gain an understanding of the experiences of mental health problems to inform recommendations on reducing stigma and increasing access to help seeking and care.

METHODS

Setting and participants

The study's sampling frame included submissions made to the It's OK to Talk web platform during the first year of its operation (April 2017 - March 2018). Eligible submissions were English-language first-person accounts of self-identified mental health problems, submitted in any media format for online publication by authors aged 18-31 years and of Indian nationality. 48 submissions from 44 authors met these criteria and were contacted for consent. An online consent form was sent to all authors by email and supplemented by a telephone call to review the study's aims, methods and data protection procedures. 33 participants gave their consent.

Ethical issues

Approval was obtained prior to commencement from the Sangath Institutional Review Board (IRB) for research with human subjects. Only submissions published on the website and therefore publicly accessible were considered for inclusion. This is in line with recommendations for the ethical conduct of internet research**[26-27]**. Participants in this study shared sensitive health information online such as their experiences of living with a mental illness. By disseminating this type of user generated content captured from publicly accessible online sources as part of a research project, there is potential to place participants at risk of harm because this content could be made identifiable in ways that were not intended by the user at the time the content was posted online[**28]**. Therefore, to further protect the identity of the participants, all personally identifiable data was removed and all direct quotations were paraphrased to ensure anonymity.

Data collection

Submissions included pieces submitted by authors in "any media form of your choice". Instructions to prospective submitters included details about language, file size, originality, and anonymity and also offered an FAQs section. Eligible submissions were downloaded from the website, and those in non-text form such as audio or video submissions were transcribed. Art works were accompanied by text by the artist contextualising their images and were analysed together.

Data analysis

We followed an integrated inductive-deductive approach to thematic content analysis**[29]**. Coders included two clinical psychologists (ESH and RS) and one public engagement specialist (PPG), with a post-doctoral researcher (JN) acting in the role of auditor. In the first instance, coders familiarised themselves with the full data set by reading and re-reading transcripts. A random subset of five submissions was independently analysed line-by-line by each coder, with a descriptive text-based code assigned to the smallest identifiable meaning units. This produced 33 initial codes which were compared and consensus was reached on a refined 'start list' of codes**[29],** grouped into 4 thematic categories with 9 sub-themes. The analysis then followed an iterative process in which coders independently applied the

For peer review only - http://bmjopen.bmj.com/site/about/guidelines.xhtml

coding framework to 10 transcripts at a time, before reaching consensus on a revised framework through comparison and discussion. The auditor reviewed the coding framework and a subset of five coded transcripts at two time points, offering additional feedback on the terminology of codes and overall structure. nVivo version 10 was used for organising and coding the data.

Patient and public involvement

After four rounds of iteration, the coding framework was reviewed by a focus group that comprised of eight study participants who were invited to volunteer and participate in the analysis. The purpose of the focus group was to engage the authors in the analysis and for them to provide quality assurance and comments to the development of the final coding framework. Participants were randomly selected from among the list of submissions originating from New Delhi where the researchers were based. They were invited by email, and all 8 invited participants attended. Based on feedback from the focus group, two further iterations of the coding framework were produced. Focus group participants were also involved in paraphrasing of quotations to be used in the manuscript from which final paraphrased quotes were selected by the coders, and on commenting on the final draft of the study manuscript. This was intended to retain their authentic voices and the depth of their narratives.

RESULTS

37 submissions by 33 authors aged 19-31 years (mean age 22 years) from 7 Indian cities (New Delhi, Lucknow, Bengaluru, Mumbai, Pune, Hyderabad and Haryana) were included for analysis. These included 24 pieces of prose (in the form of a personal essay or 'blog'), 9 poems, 3 pieces of visual art and one video, submitted by 13 male and 20 female authors. 4 participants had 2 submissions each and these were treated as individual data files for analysis. 4 participants chose to submit anonymously, not including their name but only providing age and gender. The analysis established four themes: 1) Living through difficulties; 2) Mental health in context; 3) Managing one's mental health; and 4) Breaking stigma and sharing hope. These themes were comprised of related sub-themes. The themes and sub-themes are described using paraphrased quotations.

Theme 1: Living through difficulties

This theme was the most dominant across the submissions (33/37) and comprised three sub-themes: Powerful, painful thoughts and feelings; Loneliness and isolation; and Day-to-day struggles.

Powerful, painful thoughts and feelings

Most narratives (29/37) included descriptions of intense distress, pain, suffering and hopelessness. These often emphasized the palpable embodied nature of negative thoughts and feelings as being physically overwhelming and burdensome. 5 narratives explicitly referred to suicidal thoughts and 3 spoke explicitly about self-harm.

"It was the first time when I resorted to self-inflicting physical pain as a coping mechanism. The idea was overwhelming and scary but the mental trauma was too much to handle. I couldn't think clear and grabbed my blade." (Male, 23)

A number of narratives described experiences of gradual onset of mental health problems without realising, as well as sudden moments of realisation after reaching a 'breaking point'. Many narratives described feeling powerless and without control and described additional fear, guilt, shame and blame experienced towards themselves as a result of their mental health or 'failure' to control thoughts and feelings. Many narratives spoke about a desperate desire to make sense of their experience, of asking 'why' and what this meant for their identities.

"Anxiety and depression felt like parts of me that would take over at different times. Seeing yourself as mentally unwell makes you feel helpless about how to handle it." (Male, 23)

For peer review only - http://bmjopen.bmj.com/site/about/guidelines.xhtml

Loneliness and isolation
Many narratives (20/37) spoke about intense feelings of isolation and loneliness.

"I am alone on a never ending road to nowhere. I am enthralling the darkness, which blinds, even the light." (Female, 24)

Some participants described the tension between their desire for connection while simultaneously wishing to isolate themselves. Many talked about difficulties conveying their mental health experiences to others due to a lack of trust or fear of rejection if they opened up.

"I have no idea how to talk about depression with my peers, colleagues, relatives or friends. There are times when I isolate myself completely, avoiding any communication." (Male, 25)

Day-to-day struggles
A sense of ongoing struggle was reflected in a number of the narratives (15/37). Some authors likened their experience of mental health difficulties to that of being in fight, war or battle. Others referred to feeling trapped, imprisoned, shackled or in a dungeon.

"I feel caged from within. It's exhausting to keep up with the struggle.
I am afraid I might be losing the battle. A part of me is dying everyday."
(Male, 25)

Many narratives spoke about putting on a mask or 'faking' their way through daily life. They talked about how sleeping, watching TV, smoking, binge eating or drinking became ways to fill a void or get temporary relief, with some authors highlighting these were maladaptive coping methods and in the longer-term made them feel worse.

"When I think back, this period of my life consists of bottling up difficult emotions and wearing a mask to make up for the emptiness I felt inside." (Male, 31)

Theme 2: Mental health in context
This theme was present across the majority of narratives (24/37) and comprised of two sub-themes: Personal, social and cultural context; and Knowledge, attitudes and stigma.

Personal, social and cultural context
Some narratives (14/37) described the context of their mental health difficulties as resulting from their personal histories, such as child abuse or growing up with a parent with mental health problems, while others alluded to wider societal factors and influences.

Some authors mentioned focal precipitating events such as bereavement or being in an abusive relationship.

"Somewhere in myself I did know we would get married owing to conservative families and different religious faiths. We still hoped though, or perhaps we ignored what we know and went ahead anyway, When I look back, this was one of the worst decisions. Although it might have given us a space to ourselves, it gave him the access to beat me up, and no one else knew. The worst was that it gave me no way out of that relationship."
(Female, 30)

For peer review only - http://bmjopen.bmj.com/site/about/guidelines.xhtml

Some narratives spoke about academic and career pressures and high expectations to 'achieve' held by themselves, families and people around them. They spoke of how acknowledging one's own mental health needs was seen as personal weakness, particularly within a culture focused on 'productivity' and achievement.

"I felt guilty about not working hard. I felt if I took a small break, disconnected myself or working a little less, admitting that I felt exhausted, I would be letting people down." (Female, 28)

"We grew up in a society blinded by success. Your status, your qualities, your choices, everything depends on that scorecard, that defines your life. Somewhere in this race, we just got caught in an infinite loop where we are endlessly running to meet those expectations, running from those failures, which in reality make no sense."
(Female, 28)

Some authors wrote about their own socio-economic privilege, class, caste and education and the impact this might have on their ability to access support for their mental health problems when they needed it.

"I have been fortunate enough to be able to access appropriate care for my mental health condition. I was lucky that I could take a break from work and give myself some time to get better. A majority of people struggling with mental health problems do not have this." (Female, 29)

Knowledge, attitudes and stigma
Many narratives (18/37) described a general lack of knowledge or understanding about mental health and negative and stigmatising attitudes. A number of participants expressed negative attitudes towards their own mental health or diagnosis and described struggling to come to terms with their difficulties. They wrote about how self-stigma or the fear of stigma had acted as a barrier to seeking help.

"In the beginning, I wasn't ready to accept the fact that there was something wrong, something that would make me look imperfect. I just wanted to be 'normal', like everyone else in my college." (Female, 25)

"People told me, 'just cheer up', not to use depression as an 'excuse' and that 'medicine is not the answer'." (Female, 25)

The important need for accurate information about mental health to be freely accessible so that people can identify when they have a problem and seek appropriate help was pointed to by several authors.

"Awareness and access to information needs to increase. Then many more people can identify problems they might be facing and get the right kind of help they need and in time." (Female, 29)

Theme 3: Managing one's mental health
Reflections on managing one's mental health difficulties were present in the majority of narratives (30/37) and comprised of five sub themes: Psychosocial support; Healthcare; Self-care; Hope and growth and Acceptance.

Psychosocial support
Some narratives (16/37) spoke about the importance of friends and family as barriers or facilitators to support.

"I began to truly realize the importance of having family and friends around me, people who understood me. All I needed was someone to lend me an ear, give me the space to break down." (Male, 25)

For peer review only - http://bmjopen.bmj.com/site/about/guidelines.xhtml

"It did not really help when my peers made no effort to show compassion towards me or understand what I was going through." (Male, 18)

Awareness by family and friends was seen as connected to their willingness and ability to offer meaningful support.

"It became my job to educate my family about my condition. They are nice people, they care about me but I could see through their helpless eyes, they were clueless about how to help me…" (Female, 29)

"My sister has been my pillar of strength. She noticed a clear change in my behaviour and actions and offered to get me to seek psychiatric help." (Male, 31)

Healthcare

Across many narratives (21/37), there were mixed experiences of mental healthcare services including visiting a psychologist or psychiatrist, being in hospital or taking medication. Some narratives pointed to the effort and courage necessary to access services in the first place, and that finding the right diagnosis or treatment takes time. Several authors described professional help as having been pivotal to their recovery.

"Therapy taught me to notice my ways of thinking and responding to things. I learned ways to break unhelpful patterns and to build on the helpful ones." (Male, 28)

A number of narratives described positive experiences of prescribed psychotropic medication.

"When I started taking medication I felt calm for the first time in my life. It was like I had been stumbling around in the darkness and suddenly it was light. The voices I heard finally were quiet." (Female, 25)

Some participants who found medication effective nevertheless expressed a reluctance or dislike of taking medication and a desire not to do so in the future. Others described negative experiences of medication and of being medicated against their will.

"My voices didn't like the meds. While they made my life hard, at least I felt something. Being dragged to the psychiatrist felt like a punishment when I had done nothing wrong." (Female, 23)

Self-care

Some participants (12/37) shared experiences of self-care methods which helped them to maintain their well-being. These included taking care of their physical health through healthy eating, exercise and sleep; making time for fun activities and relaxation; making changes to their relationship with work or study; and finding ways to express themselves through creative activities or talking to others.

"I have made changes in my office, making time in our meetings to talk about how we are feeling. It was a bit strange at first, but it soon became a vital part of how we worked together and supported each other. (Female, 28)"

Hope and growth

Many narratives (19/37) described experiences of recovery or improvements in mental health. Some noted progress in relationships and achievements in their education or careers accomplished in spite of mental health difficulties. Some authors conveyed the importance of holding on to hope through the difficult times.

For peer review only - http://bmjopen.bmj.com/site/about/guidelines.xhtml

"I still have my bad days but I am better at dealing with them now. True happiness still feels like a distant dream and thoughts about my future worry me. But I know I shall get there someday." (Female, 22)

Several participants spoke about personal growth through the experiences of mental health problems.

"Experiencing a mental disorder taught me a lot about my own self. It made me grow and become stronger. It was the beginning of a new journey. I got back to what I loved doing, painting and writing!" (Female, 19)

Acceptance
Many narratives (15/37) described a journey towards self-awareness and acceptance of themselves and their mental health difficulties. Acceptance of future setbacks was also noted.

"I have started finding reassurance in the way I am able to deal with the ups and downs. I am more forgiving and understanding with myself; I know there will be challenges, it's only natural, it's part of the journey." (Male, 23)

Theme 4: Breaking stigma and sharing hope
The majority of narratives (22/37) contained messages of hope directed at a wider audience and expressed the desire to break stigma. Within these messages two sub-themes were identified; It's only human and Importance of talking and listening.

It's only human!
Some narratives (8/37) emphasised that '*you are not alone*' and spoke about the importance of understanding mental health difficulties as part of being human. Many pointed towards the equivalence of mental and physical health as a way to normalise mental health difficulties.

"Mental illness is actually no different than a physical illness. It is just the same as having a cold, or a fracture or any physically manifested disease that you see a doctor for." (Female, 26)
Importance of talking and listening
The importance of mental health dialogue both personally and more broadly in society came out across many narratives (13/37). Participants offered suggestions and advice to future caregivers or friends emphasising listening and acceptance as being among the most important things they could offer someone in need.

"We must talk about mental health at every level - in schools, at work, with medical professionals and in the media. We need more information and awareness so that people can spot problems and get help. It's not just OK to talk, it is absolutely essential!" (Female, 29)

DISCUSSION
This study set out to explore the experiences of mental health problems of young Indians through a content analysis of their submissions to a mental health website. Four overarching themes were identified of 1) Living through difficulties; 2) Mental health in context; 3) Managing one's mental health; and 4) Breaking stigma and sharing hope. Almost all submissions contained personal experiences of distress with the embodied experience of mental health difficulties as physically and mentally overwhelming and burdensome. Loneliness, isolation and a strong desire for connection were prominent and these categories mirror findings of other qualitative studies of youth narratives from the UK and Americas in which themes of *'I am powerless'* and *'I am utterly alone'* were found**[24,30]**.

The need to express and share these painful experiences appears to be a primary use of the It's Ok To Talk website. This is consistent with evidence that online spaces are commonly used by people with

For peer review only - http://bmjopen.bmj.com/site/about/guidelines.xhtml

stigmatised health conditions for personal expression and social connection**[17-18]**. Informality and anonymity of the internet is emphasized in the extent to which it reduces the need to self-identify**[14]** and youth consulted during the design of the It's Ok To Talk website also felt that most submitters might not wish to disclose their identities. However though the website offered anonymity, most (89% in this study) participants chose to include their name, age and location. This could be a reflection of the strong desire for disclosure, connection and to break stigma.

Some participants described their mental health difficulties within the context of broader socio-economic and cultural contexts. In particular, how rigid social norms including those focused on academic performance, socio-economic class or religion can result in oppressive and unrealistic expectations, in turn exacerbating stress and poor mental health. In India, academic failure and stress have been noted in verbal autopsies of young people and as a predictor of suicidal ideation**[31]**.This is also consistent with other research in developing countries which has found academic as well as social problems including interpersonal and family difficulties to be among the key social determinants of poor mental health and suicide in young people**[4, 32-33**]. Submitters are not just using the It's Ok To Talk website as a way to talk about their internal experiences but to explore and challenge the societal and political contexts of their difficulties, promoting a richer and multi-dimensional understanding of mental health.

Participants raised concerns regarding equity of access to mental health care, and acknowledged structural barriers that prevent the majority of Indians from finding or accessing care. Attitudinal barriers to help-seeking were also widely endorsed, as related to public and self-stigma. The negative impacts of stigma on help-seeking for young people have been demonstrated consistently**[34]**; in a systematic review self-stigma and treatment stigma had the most consistent negative association with help-seeking**[35]**. The influence of stigma was prominent in narratives around recovery, and particularly the finding that self-acceptance and acceptance of mental health problems and diagnosis appeared to be a central part of many participants' journeys of recovery. For some, sharing their story on the website was explicitly described as part of this process of self-acceptance. This is also consistent with literature on the impact of disclosure on self-stigma**[23]**. Research shows that selective online disclosure of potentially stigmatizing health information may increase one's self-confidence, facilitate connections with similar others online and allow individuals to feel more accepting about their condition in offline settings**[6]**. In this study, in addition to fostering acceptance, many participants discussed other parts of themselves, their lives and achievements and voiced that their mental health problems alone did not define them as a person.

Participants expressed mixed experiences of mental healthcare. In contrast to the bloggers studied by Marcus et al (2012), the young people in this study were largely positive about their experiences of mental health professionals, and made recommendations to others about seeking professional help. Expressed attitudes towards medication however were mostly negative, even for those who found it helpful. It appears that young people's attitudes about psychotropic drugs are complex and influenced by a combination of factors ranging from personal experiences, fears of side effects and individual values and preferences. Negative attitudes or beliefs about taking medication may in turn be influenced by broader societal stereotypes, stigmas or misinformation (for example, "Having to take medication for my mental health means I am not trying hard enough"). There is evidence of possible links between stigma and discontinuation of medication for mental health problems**[36-38].**

A strong personal desire to break stigma, feel more confident and reduce their own self-stigma was expressed by many participants. The need for better information and understanding of mental health was seen as central to this process. Relatedly, participants commonly emphasised the importance of bringing out mental health dialogue in all social spaces; from homes, educational institutions and workplaces to public places and in communities. Some participants specifically stated this as their personal motivation for submitting their story. We note similar findings from other research showing a growing acceptance

For peer review only - http://bmjopen.bmj.com/site/about/guidelines.xhtml

that online communities can serve as powerful venues for people to challenge stigma through personal empowerment and by providing hope to others**[15].**

Limitations

Limitations of this study included a convenience sample resulting in a narrow demographic cohort of self-selected, highly educated, English speakers (the website is in English) with access to the internet or social media. Hence, users are unlikely to be representative of the general youth population in India, which is a middle-income country with large disparities in access to internet and social media**[39]**. This is also consistent with US data that show individuals who openly share illness experiences online are more highly educated than general population**[40]**. A further limitation is that the mental health problems written about were self-reported and could not be differentiated into diagnostic or other clinical categories. Moreover, it is important to note that the findings in this study do not tell us about possible benefits or direct impacts of online narratives on wellbeing or mental health for either authors or readers of the website. Further research is needed to explore this important question.

Although there are limitations in this study, we believe it adds to the growing body of knowledge on digital communication and personal narratives, and how these can play a role in addressing the needs of young people living with mental health problems. The assumption underlying the website initiative, that communicating the experience of mental health may provide positive psychosocial benefits to authors themselves as well as readers, appears to be supported. Efforts are required to determine whether similar online spaces can be used successfully by young people from lower socioeconomic groups, with lower education levels, from rural areas, and from underrepresented or marginalized population subgroups. The launch of a Hindi version of the website is planned for 2019, with the aim to extend the reach of the site to include the vast majority of young internet users in India whose language of use is Hindi.

Implications

Social media offers opportunities for young people to share personal narratives without temporal or spatial barriers**[12]**. Future research is needed to expand on the exploratory work described here to assess the effects of self-disclosure through online narratives both for promoting the mental health and wellbeing of those who have made these disclosures, and on attitudes regarding mental health problems in the wider community.

It is possible that the process of communicating the experience of mental health and distress through blogging and other media may be a way of assisting young people with mental health problems to express themselves and provide important content for authentic anti-stigma messages. The personal narratives offer a window into young people's priorities and challenges in their own words and offer more specific and nuanced key messages for awareness and help-seeking for future anti-stigma public engagement efforts, both online and through on-ground efforts.

"Listen, don't try to solve the problem."

"If you're struggling, know that it isn't your fault."

"You need to educate yourself so you can help others."

"There's no wrong time to get help."

"The most important thing to remember is that you're not alone."

For peer review only - http://bmjopen.bmj.com/site/about/guidelines.xhtml

For peer review only

For peer review only - http://bmjopen.bmj.com/site/about/guidelines.xhtml

CONTRIBUTORSHIP

1. Pattie Pramila Gonsalves: Research design, Coder, Lead author
2. Dr Eleanor Sara Hodgson: Research design, Coder, Second author
3. Rhea Sharma: Coder
4. Sweta Pal: Data management
5. Dr John Naslund: Research design, auditor, reviewing manuscript
6. Dr Daniel Michelson: Reviewing and revising manuscript
7. Dr Vikram Patel: Research design, reviewing and revising manuscript

ACKNOWLEDGEMENTS

The authors would like to thank all the participants who provided their valuable time to share their experiences and contribute to the coding and review of the manuscript. They are also grateful to Sunanda Jalote and Siddharth Parambi for their contributions.

FUNDING

This project is supported by the Wellcome Trust, UK.

ETHICS APPROVAL

Sangath Institutional Review Board (IRB)

DATA SHARING

Additional information is available by emailing pattie.gonsalves@sangath.in

For peer review only

REFERENCES

1. United Nations Population Fund (UNFPA). The power of 1.8 billion: adolescents, youth and the transformation of the future. 2014;5 https://www.unfpa.org/sites/default/files/pub-pdf/EN-SWOP14-Report_FINAL-web.pdf (accessed 26 Sep 2018).

2. Patel V, Saxena S, Lund C et al. The Lancet Commission on global mental health and sustainable development. *The Lancet* Published Online First: 2018. doi:10.1016/s0140-6736(18)31612-x

3. Patel V, Saxena S, Frankish H, et al. Sustainable development and global mental health—a Lancet Commission. Lancet 2016;378(10024):1143-1145. doi: https://doi.org/10.1016/S0140-6736(16)00208-7

4. Patel V, Ramasundarahettige C, Vijayakumar L, et al. Suicide mortality in India: a nationally representative survey. Lancet 2012;379(9834):2343-2351. doi:10.1016/S0140-6736(12)60606-0.

5. Ziebland S, Wyke S. Health and Illness in a Connected World: How Might Sharing Experiences on the Internet Affect People's Health?. Milbank Quarterly 2012;90:219-249. doi:10.1111/j.1468-0009.2012.00662.x

6. Naslund J, Aschbrenner K, Marsch L et al. The future of mental health care: peer-to-peer support and social media. Epidemiology and Psychiatric Sciences 2016;25:113-122. doi:10.1017/s2045796015001067

7. Kemp S. Digital in 2017: Global Overview - We Are Social. We Are Social. 2018.https://wearesocial.com/special-reports/digital-in-2017-global-overview (accessed 26 Sep 2018).

8. Patel V, Flisher A, Hetrick S et al. Mental health of young people: a global public-health challenge. The Lancet 2007;369:1302-1313. doi:10.1016/s0140-6736(07)60368-7

9. United Nations Children's Fund (UNICEF). The State of the World's Children 2017: Children in a Digital World. UNICEF. 2017.https://www.unicef.org/publications/index_101992.html (accessed 26 Sep 2018).

10. Brody H. Stories of Sickness. New Haven, CT: Yale University Press. 1987.

11. Shah C. Social Media and Social Networking. *Social Information Seeking* 2017;:29-42. doi:10.1007/978-3-319-56756-3_3

12. Betton V, Borschmann R, Docherty M et al. The role of social media in reducing stigma and discrimination. British Journal of Psychiatry 2015;206:443-444. doi:10.1192/bjp.bp.114.152835

13. Kaplan A, Haenlein M. Users of the world, unite! The challenges and opportunities of Social Media. Business Horizons 2010;53:59-68. doi:10.1016/j.bushor.2009.09.003

For peer review only

14. Berger M, Wagner T, Baker L. Internet use and stigmatized illness. Social Science & Medicine 2005;61:1821-1827. doi:10.1016/j.socscimed.2005.03.025

15. Lawlor A, Kirakowski J. Online support groups for mental health: A space for challenging self-stigma or a means of social avoidance?. Computers in Human Behavior 2014;32:152-161. doi:10.1016/j.chb.2013.11.015

16. Burns J, Durkin L, Nicholas J. Mental Health of Young People in the United States: What Role Can the Internet Play in Reducing Stigma and Promoting Help Seeking?. Journal of Adolescent Health 2009;45:95-97. doi:10.1016/j.jadohealth.2008.12.006

17. Harvey K, Brown B, Crawford P et al. 'Am I normal?' Teenagers, sexual health and the internet. Social Science & Medicine 2007;65:771-781. doi:10.1016/j.socscimed.2007.04.005

18. Naslund J, Grande S, Aschbrenner K et al. Naturally Occurring Peer Support through Social Media: The Experiences of Individuals with Severe Mental Illness Using YouTube. PLoS ONE 2014;9:e110171. doi:10.1371/journal.pone.0110171

19. Rickwood D, Deane F, Wilson C. When and how do young people seek professional help for mental health problems? 2007;Med J Aust 2007;187:S35-39.

20. Itsoktotalk.in. (2018). It's Ok To Talk. [online] Available at: http://itsoktotalk.in/ [Accessed 26 Sep. 2018].

21. World Health Organization. (2016). World Health Day 2017. [online] Available at: http://www.who.int/campaigns/world-health-day/2017/campaign-essentials/en/ [Accessed 26 Sep. 2018].

22. Corrigan P, Kosyluk K, Rüsch N. Reducing Self-Stigma by Coming Out Proud. American Journal of Public Health 2013;103:794-800. doi:10.2105/ajph.2012.301037

23. Corrigan P, Morris S, Larson J et al. Self-stigma and coming out about one's mental illness. Journal of Community Psychology 2010;38:259-275. doi:10.1002/jcop.20363

24. Marcus M, Westra H, Eastwood J et al. What Are Young Adults Saying About Mental Health? An Analysis of Internet Blogs. Journal of Medical Internet Research 2012;14:e17. doi:10.2196/jmir.1868

25. Ziebland S, McPherson A. Making sense of qualitative data analysis: an introduction with illustrations from DIPEx (personal experiences of health and illness). Medical Education 2006;40:405-414. doi:10.1111/j.1365-2929.2006.02467.x

For peer review only

26. Brownlow C, O'Dell L. Ethical Issues for Qualitative Research in On-line Communities. Disability & Society 2002;17:685-694. doi:10.1080/0968759022000010452

27. Eysenbach G, Wyatt J. Using the Internet for Surveys and Health Research. Journal of Medical Internet Research 2002;4:e13. doi:10.2196/jmir.4.2.e13

28. Williams M, Burnap P, Sloan L. Towards an Ethical Framework for Publishing Twitter Data in Social Research: Taking into Account Users' Views, Online Context and Algorithmic Estimation. Sociology 2017;51:1149-1168. doi:10.1177/0038038517708140

29. Bradley E, Curry L, Devers K. Qualitative Data Analysis for Health Services Research: Developing Taxonomy, Themes, and Theory. Health Services Research 2007;42:1758-1772. doi:10.1111/j.1475-6773.2006.00684.x

30. Midgley N, Parkinson S, Holmes J et al. Beyond a diagnosis: The experience of depression among clinically-referred adolescents. *Journal of Adolescence* 2015;44:269-279. doi:10.1016/j.adolescence.2015.08.007

31. Aaron R, Joseph A, Abraham S et al. Suicides in young people in rural southern India. The Lancet 2004;363:1117-1118. doi:10.1016/s0140-6736(04)15896-0x

32. Parikh R, Sapru M, Krishna M et al. 'It is lot like mind attack': A qualitative study of stress and coping among adolescents in India. *BMC Psychiatry* 2018. (In submission)

33. Patel V, Araya R, Chatterjee S. et al. Treatment and prevention of mental disorders in low-income and middle-income countries. The Lancet 2007;370:991-1005. doi:10.1016/s0140-6736(07)61240-9

34. Gulliver A, Griffiths K, Christensen H. Perceived barriers and facilitators to mental health help-seeking in young people: a systematic review. BMC Psychiatry 2010;10. doi:10.1186/1471-244x-10-113

35. Clement S, Schauman O, Graham T et al. What is the impact of mental health-related stigma on help-seeking? A systematic review of quantitative and qualitative studies. Psychological Medicine 2014;45:11-27. doi:10.1017/s0033291714000129

36. Sirey J, Bruce M, Alexopoulos G et al. Perceived Stigma as a Predictor of Treatment Discontinuation in Young and Older Outpatients With Depression. American Journal of Psychiatry 2001;158:479-481. doi:10.1176/appi.ajp.158.3.479

37. Corrigan P. How stigma interferes with mental health care. *American Psychologist* 2004;59:614-625. doi:10.1037/0003-066x.59.7.614

38. Livingston J, Boyd J. Correlates and consequences of internalized stigma for people living with mental illness: A systematic review and meta-analysis. Social Science & Medicine 2010;71:2150-

For peer review only - http://bmjopen.bmj.com/site/about/guidelines.xhtml

2161. doi:10.1016/j.socscimed.2010.09.030

39. Internet access grows worldwide but remains higher in advanced economies. Pew Research Center's Global Attitudes Project. 2018. http://www.pewglobal.org/2016/02/22/internet-access-growing-worldwide-but-remains-higher-in-advanced-economies/ (Accessed 11 Oct 2018).

40. Naslund J, Aschbrenner K, Araya R et al. Digital technology for treating and preventing mental disorders in low-income and middle-income countries: a narrative review of the literature. *The Lancet Psychiatry* 2017;4:486-500. doi:10.1016/s2215-0366(17)30096-2/

For peer review only

For peer review only - http://bmjopen.bmj.com/site/about/guidelines.xhtml

Consolidated criteria for reporting qualitative studies (COREQ): 32-item checklist

No	Item	Description	Location in the manuscript (Section, pg no.)
**Domain 1: Research team and reflexivity**			
Personal Characteristics			
1.	Interviewer/facilitator	NA	NA
2.	Credentials	PPG: MSc<br>ESH: DClinPsy<br>RS: MSc<br>JN: PhD	Title page,1
3.	Occupation	PPG, ESH and RS are presently employed with Sangath, a mental health research NGO.<br>JN is presently a Post-Doc candidate at Harvard Medical School.	Title page,1
4.	Gender	PPG, ESH, RS: Female<br>JN: Male	NA
5.	Experience and training	PPG and RS had acquired training on qualitative research in MSc degree programs and have more than 3 years of research experience including qualitative research.<br>ESH is a clinical psychologist with training in qualitative research design and practice. She has previously conducted and published qualitative research.<br>JN has extensive experience with qualitative analysis.	Title page, Methods, 5-6
Relationship with participants			
6.	Relationship established	Yes	Methods, 6
7.	Participant knowledge of the interviewer	Study participants and FGD participants were briefed about the purpose of the study and written	Methods, 6

For peer review only

		information was also provided by email and telephone. The necessary ethical approvals were received before commencement of study.	
8.	Interviewer characteristics	There are no potential sources of bias.	NA
**Domain 2: study design**			
Theoretical framework			
9.	Methodological orientation and Theory	We followed an integrated inductive-deductive approach to thematic content analysis	Methods,
Participant selection			
10.	Sampling	The study's sampling frame included submissions made to a web platform during the first year of its operation. Participants involved in a small FGD and paraphrasing activities were randomly selected from among the list of submissions originating from New Delhi where the researchers were based.	Methods, 5-6
11.	Method of approach	All prospective participants to the analysis and the FGD were approached by email.	Methods, 6
12.	Sample size	33	Methods, 5
13.	Non-participation	11	Methods, 5
Setting			
14.	Setting of data collection	The study's sampling frame included submissions made to a web platform during the first year of its operation.<br>The small participant FGD was conducted at the office premises of the researchers.	Methods, 8

For peer review only

15.	Presence of non-participants	No	
16.	Description of sample	The study's sampling frame included submissions made to the It's OK to Talk web platform during the first year of its operation (April 2017 - March 2018). Eligible submissions were English-language first-person accounts of self-identified mental health problems, submitted in any media format for online publication by authors aged 18-31 years and of Indian nationality. 37 submissions by 33 authors aged 19-31 years (mean age 22 years) from 7 Indian cities (New Delhi, Lucknow, Bengaluru, Mumbai, Pune, Hyderabad and Haryana) were included for analysis.	Methods, 5
Data collection			
17.	Interview guide	The data included in the analysis was guided only by the information on the website to share stories of mental health and raise awareness about youth mental health problems.<br><br>Yes, the interview guide for the FGD was prepared by the authors for the purpose of this study.	Introduction, 4<br>Methods, 8
18.	Repeat interviews	No	-
19.	Audio/visual recording	No	-
20.	Field notes	Field notes were made during the FGD	Methods, 6
21.	Duration	The FGD lasted 90 minutes	Methods, 6
22.	Data saturation	NA	-
23.	Transcripts returned	NA	-
**Domain 3: analysis and**			

For peer review only

**findings**			
Data analysis			
24.	Number of data coders	3 coders, 1 auditor	Methods, 5-6
25.	Description of the coding tree	Deductive and inductive codes were developed. Related codes were ordered into meaningful categories to convey interrelated ideas.	Methods, 5-6
26.	Derivation of themes	Yes	Methods, 5-6
27.	Software	nVivo 10	Methods, 6
28.	Participant checking	Focus group participants provided quality assurance and feedback on the analysis	Methods, 6
Reporting			
29.	Quotations presented	All personally identifiable data was removed and all direct quotations were paraphrased to ensure anonymity.	Results, 6-11
30.	Data and findings consistent	Yes	Results, 6-11
31.	Clarity of major themes	Yes	Results, 6-11
32.	Clarity of minor themes	Yes	Results, 6-11

For peer review only

BMJ Open

What are young Indians saying about mental health? A content analysis of blogs on the It's Ok To Talk website

Journal:	*BMJ Open*
Manuscript ID	bmjopen-2018-028244.R1
Article Type:	Research
Date Submitted by the Author:	08-Mar-2019
Complete List of Authors:	Gonsalves, Pattie; Sangath,<br>Hodgson , Eleanor ; Sangath<br>Michelson, Daniel ; University of Sussex, School of Psychology<br>Pal, Sweta ; Sangath<br>Naslund, John; Harvard Medical School, Department of Global Health and Social Medicine<br>Sharma, Rhea; Sangath<br>Patel, Vikram; Harvard Medical School, Department of Global Health and Social Medicine
**Primary Subject Heading**:	Mental health
Secondary Subject Heading:	Mental health, Public health, Qualitative research, Global health
Keywords:	MENTAL HEALTH, QUALITATIVE RESEARCH, Child & adolescent psychiatry < PSYCHIATRY

SCHOLARONE™
Manuscripts

Title of the article
What are young Indians saying about mental health? A content analysis of blogs on the It's Ok To Talk website

Full name, postal address, e-mail and telephone number of the corresponding author:
Pattie P. Gonsalves
Sangath, C-1/52, First Floor, SDA, New Delhi, India
pattie.gonsalves@sangath.in
91.9899419704

Full name, department, institution, city and country of all co-authors:

	Full Name	*Department*	*Institution*	*City*	*Country*
1	Pattie Pramila Gonsalves	PRIDE Project	Sangath	New Delhi	India
2	Dr Eleanor Sara Hodgson	PRIDE Project	Sangath	New Delhi	India
3	Dr Daniel Michelson	School of Psychology	University of Sussex	Brighton	UK
4	Sweta Pal	PRIDE Project	Sangath	New Delhi	India
5	Dr John Naslund	Department of Global Health and Social Medicine	Harvard Medical School	Boston	USA
6	Rhea Sharma	PRIDE Project	Sangath	New Delhi	India
7	Dr Vikram Patel	Department of Global Health and Social Medicine	Harvard Medical School	Boston	USA

Word count, excluding title page, abstract, references, figures and tables: 4732

Keywords: Young people; mental health; social media; qualitative research

For peer review only - http://bmjopen.bmj.com/site/about/guidelines.xhtml

ABSTRACT

Objectives: This study used thematic content analysis to examine submissions to a youth mental health website, www.itsoktotalk.in, in India.

Setting: We considered submissions made to the It's OK to Talk web platform during the first year of its operation (April 2017 - March 2018), focusing specifically on website users based in India.

Participants: We analysed 37 submissions by 33 authors aged 19-31 years (mean age 22 years) from 7 Indian cities (New Delhi, Lucknow, Bengaluru, Mumbai, Pune, Hyderabad and Haryana). Eligible submissions were English-language first-person accounts of self-identified mental health problems, submitted in any media format for online publication by authors aged 18 years or older and who were based in India. Eight study participants were additionally involved in a focus group that contributed to the coding process and preparation of the final manuscript.

Results: Four themes were identified:1) Living through difficulties; 2) Mental health in context; 3) Managing one's mental health; and 4) Breaking stigma and sharing hope. Overall, the participants expressed significant feelings of distress and hopelessness as a result of their mental health problems; many described the context of their difficulties as resulting from personal histories or wider societal factors; a general lack of understanding about mental health; and widespread stigma and other negative attitudes. Most participants expressed a desire to overcome mental health prejudice and discrimination.

Conclusions: Personal narratives offer a window into young people's self-identified priorities and challenges related to mental health problems and recovery. Such insights can inform anti-stigma initiatives and other public awareness activities around youth mental health.

ARTICLE SUMMARY

Strengths and limitations of the study

- Strengths
  - anonymised, first-person accounts of mental health problems and recovery offer the potential to illuminate issues that may not arise in other contexts (e.g. clinical encounters)
  - participatory study design (involving youth participants in the coding process and manuscript preparation) may have benefits for the quality of research
- Limitations
  - convenience sampling may have resulted in a narrow demographic cohort of self-selected, highly educated, English speakers
  - narrative descriptions of mental health problems could not be differentiated into diagnostic or other clinical categories

For peer review only

INTRODUCTION

"I hope that reading my story might help others feel less alone. It is important to know that other people are fighting the same battles. This is for anyone who feels stuck, overwhelmed or hopeless. You are not alone, just try to hold on." - (Author, female, 24, www.itsoktotalk.in)

India has the world's largest population of young people ages 10-24**[1]**, and mental health problems are the leading health concern for this group. The negative impacts of youth mental health problems are starkly reflected in the strong association between poor adolescent mental health and long-term social disability, while suicide is the leading cause of death for 15-24 year-olds in India**[2-4]**. There is growing interest in the use of social media as a vehicle for mental health promotion and stigma reduction at a population level, particularly among young people. This reflects the developmental significance of peer influence in the adolescent years and the rapid uptake of internet and mobile technology by young people across the globe**[1, 5]**. Online social networks may also be harnessed to support recovery from mental problems**[6]**. Such opportunities are especially relevant in India where rapidly increasing numbers of young people are becoming connected to technology and social media**[7]**. At the same time, fewer than 10% of young Indians have access to formal mental health services, highlighting an urgent need to identify innovative strategies to promote mental health for this age group nationally and in low- and middle-income countries more generally**[8-9]**.

Sharing mental health stories online

*"Stories are essential as a means of how scientific knowledge, in its generality, can be applied to individuals in their particularity."***[10]**

"Social media" refers to websites and applications that enable users to create and share content or participate in social networking**[11]**. Social media platforms are inexpensive and relatively easy to use, enabling individuals to bring personal experiences into the public domain and thereby impact attitudes among larger groups**[12-13]**. Betton et. al. (2015) argue that individuals, rather than institutions, are leading the way in bringing mental health conversations into online spaces, particularly through social media. Individuals with stigmatised illnesses often avoid seeking healthcare**[14]**. For such groups, reading or hearing about the experiences of others can challenge stigma**[15]** and affect health-related decision making and adjustment to an illness**[5]**. Young people with mental health difficulties report that one of the primary reasons for connecting with others online is to feel less alone**[16-18]**. Research also shows that young people may be more inclined to seek help for their mental health if they feel able to express their feelings, as well as being equipped with knowledge about mental health issues and available sources of support**[19]**.

About "It's Ok To Talk"

The It's Ok To Talk website (www.itsoktotalk.in)**[20]** was launched on WHO World Health Day (7th April) 2017 as part of a public engagement initiative by the Indian NGO Sangath. It took inspiration from the theme for that year, "Let's Talk", which emphasized the central role of disclosure in recovery from depression**[21]**. There is also evidence that disclosing a mental health problem can reduce self-stigma**[22-23]**. The website was designed in consultation with young people aged 15-24 years in New Delhi originally as an English-lanugage social media platform for young people to share their stories of mental health problems and recovery. Authors were given the choice to submit any kind of media and publish anonymously or to include their name, location and gender. All submissions required an email address and optionally a phone number for administrative purposes.

About this study

Comparatively few studies have explored first-person accounts of the lived experience of youth mental health difficulties**[24]**. Such self-directed narratives may provide rich insights that are not available in other contexts (e.g. clinical encounters)**[25]**. The current study aimed to understand the firsthand experiences of mental health problems based on information shared by young Indians through the website www.itoktotalk.in. An important feature of the research was the participation of website users in the coding of qualitative data and preparation of the final manuscript. These activities ensured that the reported results would be grounded in the lived experience of young people.

METHODS

Setting and participants

The study's sampling frame included submissions made to the It's OK to Talk website during the first year of its operation (April 2017 - March 2018). Eligible submissions were English-language, first-person accounts of self-identified mental health problems, submitted in any media format for online publication by authors aged 18-31 years and of Indian nationality. Forty-eight submissions from 44 authors met these criteria and were contacted for consent. An online consent form was sent to all authors by email and supplemented by a telephone call to review the study's aims, methods and data protection procedures. Thirty-three participants gave their consent; 11 authors did not respond to two email reminders and three phone call attempts.

Ethical issues

Approval was obtained prior to commencement from the Sangath Institutional Review Board (IRB) for research with human subjects. Only submissions published on the website and therefore publicly accessible were considered for inclusion. This is in line with recommendations for the ethical conduct of internet research**[26-27]**. Personally identifiable data were removed and all direct quotations were paraphrased to ensure anonymity.

Data collection

Instructions to prospective authors elicited submissions in "any media form of your choice", along with further details (supported by FAQs) about language, file size, originality, and anonymity. The instructions also requested that visual art submissions should be accompanied by text that contextualised the image(s). This written content was used as the basis for interpreting and coding the artwork submissions. All submissions were downloaded from the website, and those containing spoken text (e.g. in audio or video submissions) were transcribed.

Data analysis

We followed an integrated inductive-deductive approach to thematic content analysis**[28]**. Coders included two clinical psychologists (ESH and RS) and one public engagement specialist (PPG), with a post-doctoral researcher (JN) acting in the role of auditor; two coders were Indian nationals (PPG and RS). In the first instance, coders familiarised themselves with the full data set by reading and re-reading transcripts. A random subset of five submissions was independently analysed line-by-line by each coder, with descriptive text-based codes assigned to the smallest identifiable meaning units. This produced 33 initial codes which were compared; consensus was reached on a refined 'start list' of codes**[28],** grouped into 4 themes with 9 sub-themes. The analysis then followed an iterative process in which coders independently applied the coding framework to 10 transcripts at a time, before reaching consensus on a revised framework through comparison and discussion. Disagreements were resolved through group discussion; had this not been possible, then decisions would have followed the majority view. Group discussions also considered potential sources of personal bias in the interpretation of the data. To this end, coders maintained personal journals in which they reflected on the influence of their own gender, race, ethnicity, nationality, caste, class and professional backgrounds.

For peer review only - http://bmjopen.bmj.com/site/about/guidelines.xhtml

The auditor reviewed the emergent coding framework by scrutinising five coded transcripts at two time points, offering additional feedback on the nomenclature and hierarchical structure of codes. During the preparation of the manuscript, senior researchers (DM, VP) reviewed drafts and provided comments, which resulted in minor changes to the coding structure and descriptive labels. nVivo version 10 was used for organising and coding the data.

Patient and public involvement

After four rounds of iteration, a focus group was held to provide quality assurance and comments on the coding framework. Participants were randomly selected from among the list of submissions originating from New Delhi where the researchers were based. Eight young people were invited by email, all of whom agreed to participate. The group reviewed the coding framework as applied to several text excerpts and provided suggestions about how to paraphrase direct quotes from selected blogs. The intention was to retain intended meaning while ensuring anonymity. Participants highlighted issues regarding terminology such as "treatment", which was considered to have negative connotations (e.g. framing mental health problems as an "illness"). Coders subsequently produced two further iterations of the coding framework, which included changes informed by participant feedback such as renaming the "treatment" theme as "healthcare". In addition, focus group participants independently paraphrased quotations to be used in the manuscript. They were also invited to review and comment on a draft of the study manuscript.

RESULTS

Data were extracted from 37 submissions by 33 authors (13 male; 20 female) aged 19-31 years (mean age 22 years) from 7 Indian cities (New Delhi, Lucknow, Bengaluru, Mumbai, Pune, Hyderabad and Haryana). These included 24 pieces of prose (in the form of a personal essay or 'blog'), nine poems, three pieces of visual art and one video. Four participants had two submissions each and these were treated as individual data files for analysis. The analysis established four themes: 1) "Living through difficulties"; 2) "Mental health in context"; 3) "Managing one's mental health"; and 4) "Breaking stigma and sharing hope". Corresponding sub-themes and illustrative quotes are presented below under each theme, along with frequency counts reflecting coverage of sub-themes across submissions.

Theme 1: Living through difficulties

This theme was comprised of three sub-themes: "Powerful, painful thoughts and feelings"; "Loneliness and isolation"; and "Mental health as a daily struggle".

Powerful, painful thoughts and feelings (29/37)

Most narratives included descriptions of intense distress, pain, suffering and hopelessness. These often emphasized the palpable embodied nature of negative thoughts and feelings as being physically overwhelming and burdensome. Five narratives explicitly referred to suicidal thoughts and three spoke explicitly about self-harm.

"It was the first time when I resorted to self-inflicting physical pain as a coping mechanism. The idea was overwhelming and scary but the mental trauma was too much to handle. I couldn't think clear and grabbed my blade." (Male, 23)

A number of narratives described experiences of gradual onset of mental health problems, as well as sudden moments of realisation after reaching a "breaking point". Many narratives described feeling powerless and without control and described additional fear, guilt, shame and blame experienced towards themselves as a result of their mental health or "failure" to control thoughts and feelings. Many narratives spoke about a desperate desire to make sense of their experience, of asking "why" and what this meant for their identities.

For peer review only - http://bmjopen.bmj.com/site/about/guidelines.xhtml

"Anxiety and depression felt like parts of me that would take over at different times. Seeing yourself as mentally unwell makes you feel helpless about how to handle it." (Male, 23)

Loneliness and isolation (20/37)

Many narratives spoke about intense feelings of isolation and loneliness.

"I am alone on a never ending road to nowhere. I am enthralling the darkness, which blinds, even the light." (Female, 24)

Some participants described the tension between their desire for connection while simultaneously wishing to isolate themselves. Many talked about difficulties conveying their mental health experiences to others due to a lack of trust or fear of rejection if they opened up.

"I have no idea how to talk about depression with my peers, colleagues, relatives or friends. There are times when I isolate myself completely, avoiding any communication." (Male, 25)

Mental health as a daily struggle (15/37).

A sense of burden and day-to-day struggle with mental health problems was reflected in a number of the narratives. Some authors likened their experience to that of being in fight, war or battle while others referred to feeling trapped, imprisoned, shackled or in a dungeon. Narratives conveyed a strong sense of an ongoing effort and fatigue both in managing their mental health and keeping up with the tasks of daily life.

"I feel caged from within. It's exhausting to keep up with the struggle.
I am afraid I might be losing the battle. A part of me is dying everyday."
(Male, 25)

Many narratives spoke about putting on a mask or "faking" their way through daily life. They talked about how sleeping, watching TV, smoking, binge eating or drinking became ways to fill a "void" or achieve temporary relief, with some authors highlighting these were maladaptive coping methods which made them feel worse in the longer-term.

Theme 2: Mental health in context

This theme was comprised of two sub-themes: "Personal, social and cultural context"; and "Knowledge, attitudes and stigma".

Personal, social and cultural context (14/37)

Some young people's narratives described the personal context of their mental health difficulties in terms of child abuse or growing up with a parent with mental health problems, while others alluded to wider societal factors and influences. Several authors otherwise mentioned focal precipitating events such as bereavement or being in an abusive relationship.

"Somewhere in myself I did know we wouldn't get married owing to conservative families and different religious faiths. We still hoped though and went ahead anyway. Although it gave us a space to ourselves, it gave him the access to beat me up, and no one else knew." (Female, 30)

Some narratives spoke about academic and career pressures and high expectations to "achieve" held by themselves, family members and other people around them. They spoke about how acknowledging one's own mental health needs was seen as personal weakness, particularly within a culture focused on "productivity" and achievement.

For peer review only - http://bmjopen.bmj.com/site/about/guidelines.xhtml

"I felt guilty about not working hard. I felt if I took a small break, disconnected myself or working a little less, admitting that I felt exhausted, I would be letting people down." (Female, 28)

Some authors wrote about their own socio-economic privilege, class, caste and education and how these factors have affected their ability to access support for their mental health problems.

"I have been fortunate enough to be able to access appropriate care for my mental health condition. I was lucky that I could take a break from work and give myself some time to get better. A majority of people struggling with mental health problems do not have this." (Female, 29)

Knowledge, attitudes and stigma (18/37)
Multiple narratives described a general lack of knowledge or understanding about mental health and experiences of negative and stigmatising attitudes. Several authors identified how others' preconceived ideas about "who has mental health", what it means to be a "man" or about their own identities resulted in denial or dismissal of their experiences.

"People told me, 'just cheer up', not to use depression as an 'excuse' and that 'medicine is not the answer'." (Female, 25)

A number of participants expressed negative attitudes, shame and guilt towards their own mental health or diagnosis and described struggling to come to terms with their difficulties. For some self-stigma or perceived public stigma had acted as a barrier to seeking help.

"In the beginning, I wasn't ready to accept the fact that there was something wrong, something that would make me look imperfect. I just wanted to be 'normal', like everyone else in my college." (Female, 25)

Several authors highlighted the need for accurate information about mental health so that people can identify when they have a problem and seek appropriate help.

"Awareness and access to information needs to increase. Then many more people can identify problems they might be facing and get the right kind of help they need and in time." (Female, 29)

Theme 3: Managing one's mental health
This was comprised of three sub-themes: "Psychosocial support"; "Healthcare"; and "Self-care and recovery".

Psychosocial support (16/37)
Many narratives spoke about the key roles played by friends and family in providing emotional and practical support. Conversely, some narratives described a lack of support from their networks as being an additional burden. The importance of being able to talk with others about their mental health, and to be heard and not to be judged, was expressed strongly across young people's narratives.

"I began to truly realize the importance of having family and friends around me, people who understood me. All I needed was someone to lend me an ear, give me the space to break down." (Male, 25)

Other authors described positive experiences of friends and family encouraging and enabling them to access professional help. Awareness and knowledge about mental health by family and friends was seen as connected to their willingness and ability to offer meaningful support.

For peer review only - http://bmjopen.bmj.com/site/about/guidelines.xhtml

"It became my job to educate my family about my condition. They are nice people, they care about me but I could see through their helpless eyes, they were clueless about how to help me…" (Female, 29)

Healthcare (21/37)
Young people described varied experiences of mental healthcare, ranging from outpatient sessions with psychologists and/or psychiatrists to hospital admissions. Some narratives pointed to the effort and courage needed to access mental health services in the first place, and that finding the right diagnosis or treatment takes time. Several authors described professional help as having been pivotal to their recovery.

"Therapy taught me to notice my ways of thinking and responding to things. I learned ways to break unhelpful patterns and to build on the helpful ones." (Male, 28)

A number of narratives described positive experiences of prescribed psychotropic medication.

"When I started taking medication I felt calm for the first time in my life. It was like I had been stumbling around in the darkness and suddenly it was light. The voices I heard finally were quiet." (Female, 25)

Some participants who found medication effective nevertheless expressed a reluctance or dislike of taking medication and a desire not to do so in the future. Others described negative experiences of medication and of being medicated against their will.

"My voices didn't like the meds. While they made my life hard, at least I felt something. Being dragged to the psychiatrist felt like a punishment when I had done nothing wrong." (Female, 23)

Self-care and recovery (26/37)
Many narratives described experiences of recovery or improvements in mental health. Some participants shared experiences of self-care methods which helped them to maintain their well-being. These included taking care of their physical health; making changes to their relationship with work or study; and finding ways to express themselves through creative activities or talking to others.

"I have made changes in my office, making time in our meetings to talk about how we are feeling. It was a bit strange at first, but it soon became a vital part of how we worked together and supported each other. (Female, 28)"

Many narratives described a journey towards self-awareness and acceptance of themselves and their mental health difficulties as central to their recovery. Some authors conveyed the importance of holding on to hope through the difficult times and an acceptance that the future would contain ups and downs.

"I still have my bad days but I am better at dealing with them now. True happiness still feels like a distant dream and thoughts about my future worry me. But I know I shall get there someday." (Female, 22)

Some authors note progress in relationships and achievements in their education or careers accomplished in spite of mental health difficulties. Others spoke about personal growth and learning achieved directly as a result of the experiences of mental health problems. Across the narratives authors described their identity as not being limited by their mental health or illness.

"Experiencing a mental disorder taught me a lot about my own self. It made me grow and become stronger. It was the beginning of a new journey. I got back to what I loved doing, painting and writing!" (Female, 19)

Theme 4: Breaking stigma and sharing hope

For peer review only - http://bmjopen.bmj.com/site/about/guidelines.xhtml

The majority of narratives contained messages directed at readers with mental health problems as well as a broader audience. Within these messages two sub-themes were identified; “Desire to help others” and “Desire to break stigma”.

Desire to help others (18/37)
Several authors shared the intention that their own story might help others by giving them hope, courage and reassurance that “you are not alone”. Some authors spoke about positive experiences of sharing their mental health stories in their personal and professional lives and how helping others also helps them. Several authors gave specific advice to readers such as encouraging them to seek professional help. Across the narratives there was a strong desire to convey that life can get better and to encourage readers not to ‘give up’.

‘I hope I can help people to free themselves from being stuck in their own difficulties’ (Female, 20)

Desire to break stigma (14/37)
Several authors identified their motivation for sharing their story in terms of normalising mental health problems and breaking stigma. Some narratives spoke about the importance of understanding mental health difficulties as a normal part of being human; others emphasised that mental health difficulties were “real” and equally important as problems affecting physical health.

“Mental illness is actually no different than a physical illness. It is just the same as having a cold, or a fracture or any physically manifested disease that you see a doctor for.” (Female, 26)

The importance of mental health dialogue both personally and more broadly in society came out across many narratives Authors emphasised the courage required to disclose the state of ones’ own mental health and stressed the powerful impact it can have on personal recovery and breaking wider stigma.

“We must talk about mental health at every level - in schools, at work, with medical professionals and in the media. We need more information and awareness so that people can spot problems and get help. It's not just OK to talk, it is absolutely essential!” (Female, 29)

DISCUSSION
This study set out to explore the lived experiences of mental health problems and recovery from a sample of young people in India, based on mixed-media submissions to a public website. Four overarching themes were identified of 1) Living through difficulties; 2) Mental health in context; 3) Managing one's mental health; and 4) Breaking stigma and sharing hope. Almost all submissions contained personal experiences of distress with the embodied experience of mental health difficulties as physically and mentally overwhelming and burdensome. Loneliness, isolation and a strong desire for connection were prominent, mirroring findings from qualitative studies of youth narratives obtained in high-income countries**[24,29]**.

The expression and sharing of such painful experiences appeared to be an important function of the It’s Ok To Talk website. This is consistent with evidence that online spaces are commonly used by people with stigmatised health conditions for personal expression and social connection**[17-18]**. Informality and anonymity of the internet is emphasized in the extent to which it reduces the need to self-identify**[14]**. Youth consulted during the design of the It’s Ok To Talk website also felt that most submitters might not wish to disclose their identities. However, although the website offered anonymity, most participants (89% in this study) chose to include their name, age and location. This could be a reflection of the strong desire to be heard and recognised.

For peer review only - http://bmjopen.bmj.com/site/about/guidelines.xhtml

Many participants described their mental health difficulties within the context of broader socio-economic and cultural contexts. These submissions went beyond mere description of internal experiences to explore and challenge the societal and political contexts of psychological distress, offering a rich and multi-dimensional understanding of mental health. In particular, young people's narratives explained how rigid social norms can result in oppressive and unrealistic expectations, exacerbating stress and contributing to poor mental health. In India, academic failure and stress have been noted in verbal autopsies of young people and as a predictor of suicidal ideation**[30]**. This is also consistent with research in other developing countries which has found academic, interpersonal and family difficulties to be among the key social determinants of poor mental health and suicide in young people**[4, 31-32**].

Participants raised concerns regarding equity of access to mental health care, and acknowledged structural barriers that prevented the majority of Indians from finding or otherwise accessing care. Consistent with extensive previous research on the pervasive effects of mental health stigma on help-seeking, both public and self-stigma also limited young people's access to services **[33,34]**. The influence of stigma was prominent in narratives around recovery, and particularly the finding that acceptance of mental health problems appeared to be a central part of many participants' journeys of recovery. For some young people, sharing their story on the website was part of this process. This is also consistent with literature on the impact of disclosure on self-stigma**[23]**. Research has shown that selective online disclosure of potentially stigmatizing health information may increase self-confidence, facilitate connections with similar others online and allow individuals to feel more accepting about their condition in offline settings**[6]**. In this study, in addition to fostering acceptance, many participants discussed other aspects of their lives and achievements, and voiced that their mental health problems alone did not define them as a person.

Participants expressed mixed experiences of mental healthcare. In contrast to the bloggers studied by Marcus et al (2012), the young people in this study were largely positive about their experiences of mental health professionals, and made recommendations to others about seeking professional help. Considering medication specifically, attitudes were mixed and predominantly negative. There is evidence of possible links between stigma and discontinuation of medication for mental health problems**[35-37].** It would be important for future qualitative research to explore possible influences of stigma, fear of side effects and individual values on attitudes towards medication.

Participants variously expressed personal motivations to mitigate public stigma, to reduce self-stigma and to help others. For instance, to reassure others they are not alone in their struggle and normalise mental health as part of the human experience. The need for better information and understanding of mental health was seen as central to this process. Relatedly, participants emphasised the importance of broadening the mental health discourse to encompass social spaces ranging from homes, educational institutions, workplaces and community settings. Similarly, other research has indicated that online communities can serve as powerful venues for people to challenge stigma through personal empowerment and by providing hope to others**[15]**.

Limitations

Limitations of this study included a convenience sample resulting in a narrow demographic cohort of self-selected, highly educated, English speakers (the website is in English) with access to the internet. Hence, participants are unlikely to be representative of the general youth population in India, which is a middle-income country with large disparities in internet access, especially among women and individuals from lower socioeconomic status**[38]**. This is also consistent with US data showing that individuals who share illness experiences online appear to be more highly educated than the general population**[39]**. A further limitation is that young people's mental health problems were self-reported and could not be differentiated into diagnostic or other clinical categories. This study also did not include any standardised measures to understand impacts on authors or readers of the website and findings do not tell us about

For peer review only - http://bmjopen.bmj.com/site/about/guidelines.xhtml

possible benefits or risks of online narratives for authors or readers. For example, some research has identified risks associated with obtaining advice from peers with unknown credentials or setting of unrealistic expectations due to learning about others' health experiences**[40]**. Further research is needed to explore these important questions.

Notwithstanding these limitations, the study adds to the growing literature on digital communication and personal narratives, and how these can play a role in addressing the needs of young people living with mental health problems. Further efforts are required to determine whether similar online spaces can be used successfully by young people from lower socioeconomic groups, with lower education levels, from rural areas, and from underrepresented or marginalized population subgroups. The launch of a Hindi version of the It's Ok To Talk website is planned for 2019, with the aim to extend the reach of the site to include a large number of of young internet users in India whose primary computing language is Hindi.

Conclusions

Social media offers opportunities to share personal narratives without temporal or spatial barriers**[12]**. Future research is needed to expand on the exploratory work described here and assess the effects of self-disclosure through online narratives. This is important both for promoting the mental health and wellbeing of those who have made these disclosures, and on attitudes regarding mental health problems in the wider community.

Such personal narratives highlight important gaps and needs in addressing young people's mental health needs. They also offer a window into young people's priorities in their own words and provide specific themes and nuanced key messages for awareness and help-seeking for future anti-stigma public engagement efforts in India or other similar settings.

"Listen, don't try to solve the problem."

"If you're struggling, know that it isn't your fault."

"You need to educate yourself so you can help others."

"There's no wrong time to get help."

"The most important thing to remember is that you're not alone."

For peer review only - http://bmjopen.bmj.com/site/about/guidelines.xhtml

CONTRIBUTORSHIP
PPG, ESH, DM, JN and VP designed the study with the support of SP and RS and PPG, ESH and RS coded the data, JN served as an auditor, and all authors contributed to interpretation of the data. PG and ESH wrote the manuscript with support from DM, SP, JN, RS and VP. All authors gave final approval of the version to be published.

COMPETING INTERESTS
The authors declare that the research was conducted in the absence of any commercial or financial relationships that could be construed as a potential conflict of interest.

ACKNOWLEDGEMENTS
The authors would like to thank all the participants who provided their valuable time to share their experiences and contribute to the coding and review of the manuscript. They are also grateful to Sunanda Jalote and Siddharth Parambi for their contributions.

FUNDING
This project has received funding from the Wellcome Trust, UK.

ETHICS APPROVAL
Sangath Institutional Review Board (IRB)

DATA SHARING
Additional information is available by emailing pattie.gonsalves@sangath.in

For peer review only

REFERENCES

1. United Nations Population Fund (UNFPA). The power of 1.8 billion: adolescents, youth and the transformation of the future. 2014;5 https://www.unfpa.org/sites/default/files/pub-pdf/EN-SWOP14-Report_FINAL-web.pdf (accessed 26 Sep 2018).

2. Patel V, Saxena S, Lund C et al. The Lancet Commission on global mental health and sustainable development. *The Lancet* Published Online First: 2018. doi:10.1016/s0140-6736(18)31612-x

3. Patel V, Saxena S, Frankish H, et al. Sustainable development and global mental health—a Lancet Commission. Lancet 2016;378(10024):1143-1145. doi: https://doi.org/10.1016/S0140-6736(16)00208-7

4. Patel V, Ramasundarahettige C, Vijayakumar L, et al. Suicide mortality in India: a nationally representative survey. Lancet 2012;379(9834):2343-2351. doi:10.1016/S0140-6736(12)60606-0.

5. Ziebland S, Wyke S. Health and Illness in a Connected World: How Might Sharing Experiences on the Internet Affect People's Health?. Milbank Quarterly 2012;90:219-249. doi:10.1111/j.1468-0009.2012.00662.x

6. Naslund J, Aschbrenner K, Marsch L et al. The future of mental health care: peer-to-peer support and social media. Epidemiology and Psychiatric Sciences 2016;25:113-122. doi:10.1017/s2045796015001067

7. Kemp S. Digital in 2017: Global Overview - We Are Social. We Are Social. 2018.https://wearesocial.com/special-reports/digital-in-2017-global-overview (accessed 26 Sep 2018).

8. Patel V, Flisher A, Hetrick S et al. Mental health of young people: a global public-health challenge. The Lancet 2007;369:1302-1313. doi:10.1016/s0140-6736(07)60368-7

9. United Nations Children's Fund (UNICEF). The State of the World's Children 2017: Children in a Digital World. UNICEF. 2017.https://www.unicef.org/publications/index_101992.html (accessed 26 Sep 2018).

10. Brody H. Stories of Sickness. New Haven, CT: Yale University Press. 1987.

11. Shah C. Social Media and Social Networking. *Social Information Seeking* 2017;:29-42. doi:10.1007/978-3-319-56756-3_3

12. Betton V, Borschmann R, Docherty M et al. The role of social media in reducing stigma and discrimination. British Journal of Psychiatry 2015;206:443-444. doi:10.1192/bjp.bp.114.152835

13. Kaplan A, Haenlein M. Users of the world, unite! The challenges and opportunities of Social Media. Business Horizons 2010;53:59-68. doi:10.1016/j.bushor.2009.09.003

For peer review only

14. Berger M, Wagner T, Baker L. Internet use and stigmatized illness. Social Science & Medicine 2005;61:1821-1827. doi:10.1016/j.socscimed.2005.03.025

15. Lawlor A, Kirakowski J. Online support groups for mental health: A space for challenging self-stigma or a means of social avoidance?. Computers in Human Behavior 2014;32:152-161. doi:10.1016/j.chb.2013.11.015

16. Burns J, Durkin L, Nicholas J. Mental Health of Young People in the United States: What Role Can the Internet Play in Reducing Stigma and Promoting Help Seeking?. Journal of Adolescent Health 2009;45:95-97. doi:10.1016/j.jadohealth.2008.12.006

17. Harvey K, Brown B, Crawford P et al. 'Am I normal?' Teenagers, sexual health and the internet. Social Science & Medicine 2007;65:771-781. doi:10.1016/j.socscimed.2007.04.005

18. Naslund J, Grande S, Aschbrenner K et al. Naturally Occurring Peer Support through Social Media: The Experiences of Individuals with Severe Mental Illness Using YouTube. PLoS ONE 2014;9:e110171. doi:10.1371/journal.pone.0110171

19. Rickwood D, Deane F, Wilson C. When and how do young people seek professional help for mental health problems? 2007;Med J Aust 2007;187:S35-39.

20. Itsoktotalk.in. (2018). It's Ok To Talk. [online] Available at: http://itsoktotalk.in/ [Accessed 26 Sep. 2018].

21. World Health Organization. (2016). World Health Day 2017. [online] Available at: http://www.who.int/campaigns/world-health-day/2017/campaign-essentials/en/ [Accessed 26 Sep. 2018].

22. Corrigan P, Kosyluk K, Rüsch N. Reducing Self-Stigma by Coming Out Proud. American Journal of Public Health 2013;103:794-800. doi:10.2105/ajph.2012.301037

23. Corrigan P, Morris S, Larson J et al. Self-stigma and coming out about one's mental illness. Journal of Community Psychology 2010;38:259-275. doi:10.1002/jcop.20363

24. Marcus M, Westra H, Eastwood J et al. What Are Young Adults Saying About Mental Health? An Analysis of Internet Blogs. Journal of Medical Internet Research 2012;14:e17. doi:10.2196/jmir.1868

25. Ziebland S, McPherson A. Making sense of qualitative data analysis: an introduction with illustrations from DIPEx (personal experiences of health and illness). Medical Education 2006;40:405-414. doi:10.1111/j.1365-2929.2006.02467.x

For peer review only

26. Brownlow C, O'Dell L. Ethical Issues for Qualitative Research in On-line Communities. Disability & Society 2002;17:685-694. doi:10.1080/0968759022000010452

27. Eysenbach G, Wyatt J. Using the Internet for Surveys and Health Research. Journal of Medical Internet Research 2002;4:e13. doi:10.2196/jmir.4.2.e13

28. Bradley E, Curry L, Devers K. Qualitative Data Analysis for Health Services Research: Developing Taxonomy, Themes, and Theory. Health Services Research 2007;42:1758-1772. doi:10.1111/j.1475-6773.2006.00684.x

29. Midgley N, Parkinson S, Holmes J et al. Beyond a diagnosis: The experience of depression among clinically-referred adolescents. *Journal of Adolescence* 2015;44:269-279. doi:10.1016/j.adolescence.2015.08.007

30. Aaron R, Joseph A, Abraham S et al. Suicides in young people in rural southern India. The Lancet 2004;363:1117-1118. doi:10.1016/s0140-6736(04)15896-0x

31. Parikh R, Sapru M, Krishna M et al. ‘It is lot like mind attack’: A qualitative study of stress and coping among adolescents in India. *BMC Psychiatry* 2018. (In submission)

32. Patel V, Araya R, Chatterjee S. et al. Treatment and prevention of mental disorders in low-income and middle-income countries. The Lancet 2007;370:991-1005. doi:10.1016/s0140-6736(07)61240-9

33. Gulliver A, Griffiths K, Christensen H. Perceived barriers and facilitators to mental health help-seeking in young people: a systematic review. BMC Psychiatry 2010;10. doi:10.1186/1471-244x-10-113

34. Clement S, Schauman O, Graham T et al. What is the impact of mental health-related stigma on help-seeking? A systematic review of quantitative and qualitative studies. Psychological Medicine 2014;45:11-27. doi:10.1017/s0033291714000129

35. Sirey J, Bruce M, Alexopoulos G et al. Perceived Stigma as a Predictor of Treatment Discontinuation in Young and Older Outpatients With Depression. American Journal of Psychiatry 2001;158:479-481. doi:10.1176/appi.ajp.158.3.479
36. Corrigan P. How stigma interferes with mental health care. *American Psychologist* 2004;59:614-625. doi:10.1037/0003-066x.59.7.614

37. Livingston J, Boyd J. Correlates and consequences of internalized stigma for people living with mental illness: A systematic review and meta-analysis. Social Science & Medicine 2010;71:2150-2161. doi:10.1016/j.socscimed.2010.09.030

For peer review only - http://bmjopen.bmj.com/site/about/guidelines.xhtml

38. Internet access grows worldwide but remains higher in advanced economies. Pew Research Center's Global Attitudes Project. 2018. http://www.pewglobal.org/2016/02/22/internet-access-growing-worldwide-but-remains-higher-in-advanced-economies/ (Accessed 11 Oct 2018).

39. Naslund J, Aschbrenner K, Araya R et al. Digital technology for treating and preventing mental disorders in low-income and middle-income countries: a narrative review of the literature. *The Lancet Psychiatry* 2017;4:486-500. doi:10.1016/s2215-0366(17)30096-2/

40. Naslund J, Aschbrenner K, McHugo G et al. Exploring opportunities to support mental health care using social media: A survey of social media users with mental illness. *Early Intervention in Psychiatry* Published Online First: 2017. doi:10.1111/eip.12496

For peer review only

For peer review only - http://bmjopen.bmj.com/site/about/guidelines.xhtml

Consolidated criteria for reporting qualitative studies (COREQ): 32-item checklist

No	Item	Description	Location in the manuscript (Section, pg no.)
**Domain 1: Research team and reflexivity**			
Personal Characteristics			
1.	Interviewer/facilitator	NA	NA
2.	Credentials	PPG: MSc<br>ESH: DClinPsy<br>RS: MSc<br>JN: PhD	Title page,1
3.	Occupation	PPG, ESH and RS are presently employed with Sangath, a mental health research NGO.<br>JN is presently a Post-Doc candidate at Harvard Medical School.	Title page,1
4.	Gender	PPG, ESH, RS: Female<br>JN: Male	NA
5.	Experience and training	PPG and RS had acquired training on qualitative research in MSc degree programs and have more than 3 years of research experience including qualitative research.<br>ESH is a clinical psychologist with training in qualitative research design and practice. She has previously conducted and published qualitative research.<br>JN has extensive experience with qualitative analysis.	Title page, Methods, 5-6
Relationship with participants			
6.	Relationship established	Yes	Methods, 6
7.	Participant knowledge of the interviewer	Study participants and FGD participants were briefed about the purpose of the study and written	Methods, 6

For peer review only

		information was also provided by email and telephone. The necessary ethical approvals were received before commencement of study.	
8.	Interviewer characteristics	There are no potential sources of bias.	NA
**Domain 2: study design**			
Theoretical framework			
9.	Methodological orientation and Theory	We followed an integrated inductive-deductive approach to thematic content analysis	Methods,
Participant selection			
10.	Sampling	The study's sampling frame included submissions made to a web platform during the first year of its operation. Participants involved in a small FGD and paraphrasing activities were randomly selected from among the list of submissions originating from New Delhi where the researchers were based.	Methods, 5-6
11.	Method of approach	All prospective participants to the analysis and the FGD were approached by email.	Methods, 6
12.	Sample size	33	Methods, 5
13.	Non-participation	11	Methods, 5
Setting			
14.	Setting of data collection	The study's sampling frame included submissions made to a web platform during the first year of its operation.<br>The small participant FGD was conducted at the office premises of the researchers.	Methods, 8

For peer review only

15.	Presence of non-participants	No	
16.	Description of sample	The study's sampling frame included submissions made to the It's OK to Talk web platform during the first year of its operation (April 2017 - March 2018). Eligible submissions were English-language first-person accounts of self-identified mental health problems, submitted in any media format for online publication by authors aged 18-31 years and of Indian nationality. 37 submissions by 33 authors aged 19-31 years (mean age 22 years) from 7 Indian cities (New Delhi, Lucknow, Bengaluru, Mumbai, Pune, Hyderabad and Haryana) were included for analysis.	Methods, 5
Data collection			
17.	Interview guide	The data included in the analysis was guided only by the information on the website to share stories of mental health and raise awareness about youth mental health problems.<br><br>Yes, the interview guide for the FGD was prepared by the authors for the purpose of this study.	Introduction, 4<br>Methods, 8
18.	Repeat interviews	No	-
19.	Audio/visual recording	No	-
20.	Field notes	Field notes were made during the FGD	Methods, 6
21.	Duration	The FGD lasted 90 minutes	Methods, 6
22.	Data saturation	NA	-
23.	Transcripts returned	NA	-
**Domain 3: analysis and**			

For peer review only

**findings**			
Data analysis			
24.	Number of data coders	3 coders, 1 auditor	Methods, 5-6
25.	Description of the coding tree	Deductive and inductive codes were developed. Related codes were ordered into meaningful categories to convey interrelated ideas.	Methods, 5-6
26.	Derivation of themes	Yes	Methods, 5-6
27.	Software	nVivo 10	Methods, 6
28.	Participant checking	Focus group participants provided quality assurance and feedback on the analysis	Methods, 6
Reporting			
29.	Quotations presented	All personally identifiable data was removed and all direct quotations were paraphrased to ensure anonymity.	Results, 6-11
30.	Data and findings consistent	Yes	Results, 6-11
31.	Clarity of major themes	Yes	Results, 6-11
32.	Clarity of minor themes	Yes	Results, 6-11

For peer review only

BMJ Open

What are young Indians saying about mental health? A content analysis of blogs on the It's Ok To Talk website

Journal:	*BMJ Open*
Manuscript ID	bmjopen-2018-028244.R2
Article Type:	Research
Date Submitted by the Author:	25-May-2019
Complete List of Authors:	Gonsalves, Pattie; Sangath,<br>Hodgson , Eleanor ; Sangath<br>Michelson, Daniel ; University of Sussex, School of Psychology<br>Pal, Sweta ; Sangath<br>Naslund, John; Harvard Medical School, Department of Global Health and Social Medicine<br>Sharma, Rhea; Sangath<br>Patel, Vikram; Harvard Medical School, Department of Global Health and Social Medicine
**Primary Subject Heading**:	Mental health
Secondary Subject Heading:	Mental health, Public health, Qualitative research, Global health
Keywords:	MENTAL HEALTH, QUALITATIVE RESEARCH, Child & adolescent psychiatry < PSYCHIATRY, Social Media

SCHOLARONE™
Manuscripts

Title of the article
What are young Indians saying about mental health? A content analysis of blogs on the It's Ok To Talk website

Full name, postal address, e-mail and telephone number of the corresponding author:
Pattie P. Gonsalves
Sangath, C-1/52, First Floor, SDA, New Delhi, India
pattie.gonsalves@sangath.in
91.9899419704

Full name, department, institution, city and country of all co-authors:

	Full Name	*Department*	*Institution*	*City*	*Country*
1	Pattie Pramila Gonsalves	PRIDE Project	Sangath	New Delhi	India
2	Dr Eleanor Sara Hodgson	PRIDE Project	Sangath	New Delhi	India
3	Dr Daniel Michelson	School of Psychology	University of Sussex	Brighton	UK
4	Sweta Pal	PRIDE Project	Sangath	New Delhi	India
5	Dr John Naslund	Department of Global Health and Social Medicine	Harvard Medical School	Boston	USA
6	Rhea Sharma	PRIDE Project	Sangath	New Delhi	India
7	Dr Vikram Patel	Department of Global Health and Social Medicine	Harvard Medical School	Boston	USA

Word count, excluding title page, abstract, references, figures and tables: 4793

Keywords: Young people; mental health; social media; qualitative research

For peer review only - http://bmjopen.bmj.com/site/about/guidelines.xhtml

ABSTRACT

Objectives: This study used thematic content analysis to examine submissions to a youth mental health website, www.itsoktotalk.in, in India.

Setting: We considered submissions made to the It's OK to Talk web platform during the first year of its operation (April 2017 - March 2018), focusing specifically on website users based in India.

Participants: We analysed 37 submissions by 33 authors aged 19-31 years (mean age 22 years) from 7 Indian cities (New Delhi, Lucknow, Bengaluru, Mumbai, Pune, Hyderabad and Haryana). Eligible submissions were English-language first-person accounts of self-identified mental health problems, submitted in any media format for online publication by authors aged 18 years or older and who were based in India. Eight study participants were additionally involved in a focus group that contributed to the coding process and preparation of the final manuscript.

Results: Four themes were identified:1) Living through difficulties; 2) Mental health in context; 3) Managing one's mental health; and 4) Breaking stigma and sharing hope. Overall, the participants expressed significant feelings of distress and hopelessness as a result of their mental health problems; many described the context of their difficulties as resulting from personal histories or wider societal factors; a general lack of understanding about mental health; and widespread stigma and other negative attitudes. Most participants expressed a desire to overcome mental health prejudice and discrimination.

Conclusions: Personal narratives offer a window into young people's self-identified priorities and challenges related to mental health problems and recovery. Such insights can inform anti-stigma initiatives and other public awareness activities around youth mental health.

ARTICLE SUMMARY

Strengths and limitations of the study

- Strengths
  - anonymised, first-person accounts of mental health problems and recovery offer the potential to illuminate issues that may not arise in other contexts (e.g. clinical encounters)
  - participatory study design (involving youth participants in the coding process and manuscript preparation) may have benefits for the quality of research
- Limitations
  - convenience sampling may have resulted in a narrow demographic cohort of self-selected, highly educated, English speakers
  - narrative descriptions of mental health problems could not be differentiated into diagnostic or other clinical categories

For peer review only - http://bmjopen.bmj.com/site/about/guidelines.xhtml

INTRODUCTION

"I hope that reading my story might help others feel less alone. It is important to know that other people are fighting the same battles. This is for anyone who feels stuck, overwhelmed or hopeless. You are not alone, just try to hold on." - (Author, female, 24, www.itsoktotalk.in)

India has the world's largest population of young people ages 10-24**[1]**, and mental health problems are the leading health concern for this group. The negative impacts of youth mental health problems are starkly reflected in the strong association between poor adolescent mental health and long-term social disability, while suicide is the leading cause of death for 15-24 year-olds in India**[2-4]**. There is growing interest in the use of social media as a vehicle for mental health promotion and stigma reduction at a population level, particularly among young people. This reflects the developmental significance of peer influence in the adolescent years and the rapid uptake of internet and mobile technology by young people across the globe**[1, 5]**. Online social networks may also be harnessed to support recovery from mental problems**[6]**. Such opportunities are especially relevant in India where rapidly increasing numbers of young people are becoming connected to technology and social media**[7]**. At the same time, fewer than 10% of young Indians have access to formal mental health services, highlighting an urgent need to identify innovative strategies to promote mental health for this age group nationally and in low- and middle-income countries more generally**[8-9]**.

Sharing mental health stories online

*"Stories are essential as a means of how scientific knowledge, in its generality, can be applied to individuals in their particularity."***[10]**

"Social media" refers to websites and applications that enable users to create and share content or participate in social networking**[11]**. Social media platforms are inexpensive and relatively easy to use, enabling individuals to bring personal experiences into the public domain and thereby impact attitudes among larger groups**[12-13]**. Betton et. al. (2015) argue that individuals, rather than institutions, are leading the way in bringing mental health conversations into online spaces, particularly through social media. Existing research highlights a complex picture of social media's relationship with health and wellbeing outcomes demonstrating both positive and negative impacts[14]. Though public discourse on social media use often emphasizes negative impacts, some research suggests that the effects may depend on the way these platforms are used [15]. Individuals with stigmatised illnesses often avoid seeking healthcare**[16]**. For such groups, reading or hearing about the experiences of others can challenge stigma**[17]** and affect health-related decision making and adjustment to an illness**[5]**. Young people with mental health difficulties report that one of the primary reasons for connecting with others online is to feel less alone**[18-20]**. Research also shows that young people may be more inclined to seek help for their mental health if they feel able to express their feelings, as well as being equipped with knowledge about mental health issues and available sources of support**[21]**.

About "It's Ok To Talk"

The It's Ok To Talk website (www.itsoktotalk.in)**[22]** was launched on WHO World Health Day (7th April) 2017 as part of a public engagement initiative by the Indian NGO Sangath. It took inspiration from the theme for that year, "Let's Talk", which emphasized the central role of disclosure in recovery from depression**[23]**. There is also evidence that disclosing a mental health problem can reduce self-stigma**[24-25]**. The website was designed in consultation with young people aged 15-24 years in New Delhi originally as an English-language social media platform for young people to share their stories of mental health problems and recovery. Authors were given the choice to submit any kind of media and publish anonymously or to include their name, location and gender. All submissions required an email address and optionally a phone number for administrative purposes.

For peer review only - http://bmjopen.bmj.com/site/about/guidelines.xhtml

About this study

Comparatively few studies have explored first-person accounts of the lived experience of youth mental health difficulties**[26]**. Such self-directed narratives may provide rich insights that are not available in other contexts (e.g. clinical encounters)**[27]**. The current study aimed to understand the firsthand experiences of mental health problems based on information shared by young Indians through the website www.itoktotalk.in. An important feature of the research was the participation of website users in the coding of qualitative data and preparation of the final manuscript. These activities ensured that the reported results would be grounded in the lived experience of young people.

METHODS

Setting and participants

The study's sampling frame included submissions made to the It's OK to Talk website during the first year of its operation (April 2017 - March 2018). Eligible submissions were English-language, first-person accounts of self-identified mental health problems, submitted in any media format for online publication by authors aged 18-31 years and of Indian nationality. Forty-eight submissions from 44 authors met these criteria and were contacted for consent. An online consent form was sent to all authors by email and supplemented by a telephone call to review the study's aims, methods and data protection procedures. Thirty-three participants gave their consent; 11 authors did not respond to two email reminders and three phone call attempts.

Ethical issues

Approval was obtained prior to commencement from the Sangath Institutional Review Board (IRB) for research with human subjects. Only submissions published on the website and therefore publicly accessible were considered for inclusion. This is in line with recommendations for the ethical conduct of internet research**[28-29]**. Personally identifiable data were removed and all direct quotations were paraphrased to ensure anonymity.

Data collection

Instructions to prospective authors elicited submissions in "any media form of your choice", along with further details (supported by FAQs) about language, file size, originality, and anonymity. The instructions also requested that visual art submissions should be accompanied by text that contextualised the image(s). This written content was used as the basis for interpreting and coding the artwork submissions. All submissions were downloaded from the website, and those containing spoken text (e.g. in audio or video submissions) were transcribed.

Data analysis

We followed an integrated inductive-deductive approach to thematic content analysis**[30]**. Coders included two clinical psychologists (ESH and RS) and one public engagement specialist (PPG), with a post-doctoral researcher (JN) acting in the role of auditor; two coders were Indian nationals (PPG and RS). In the first instance, coders familiarised themselves with the full data set by reading and re-reading transcripts. A random subset of five submissions was independently analysed line-by-line by each coder, with descriptive text-based codes assigned to the smallest identifiable meaning units. This produced 33 initial codes which were compared; consensus was reached on a refined 'start list' of codes**[30],** grouped into 4 themes with 9 sub-themes. The analysis then followed an iterative process in which coders independently applied the coding framework to 10 transcripts at a time, before reaching consensus on a revised framework through comparison and discussion. Differing views were resolved through group discussion with all authors. Group discussions also considered potential sources of personal bias in the interpretation of the data. To this end, coders maintained personal journals in which they reflected on the influence of their own gender, race, ethnicity, nationality, caste, class and professional backgrounds.

For peer review only - http://bmjopen.bmj.com/site/about/guidelines.xhtml

The auditor reviewed the emergent coding framework by scrutinising five coded transcripts at two time points, offering additional feedback on the nomenclature and hierarchical structure of codes. During the preparation of the manuscript, senior researchers (DM, VP) reviewed drafts and provided comments, which resulted in minor changes to the coding structure and descriptive labels. nVivo version 10 was used for organising and coding the data.

Patient and public involvement

After four rounds of iteration, a focus group was held to provide quality assurance and comments on the coding framework. Participants were randomly selected from among the list of submissions originating from New Delhi where the researchers were based. Eight young people were invited by email, all of whom agreed to participate. The group reviewed the coding framework as applied to several text excerpts and provided suggestions about how to paraphrase direct quotes from selected blogs. The intention was to retain intended meaning while ensuring anonymity. Participants highlighted issues regarding terminology such as "treatment", which was considered to have negative connotations (e.g. framing mental health problems as an "illness"). Coders subsequently produced two further iterations of the coding framework, which included changes informed by participant feedback such as renaming the "treatment" theme as "healthcare". In addition, focus group participants independently paraphrased quotations to be used in the manuscript. They were also invited to review and comment on a draft of the study manuscript.

RESULTS

Data were extracted from 37 submissions by 33 authors (13 male; 20 female) aged 19-31 years (mean age 22 years) from 7 Indian cities (New Delhi, Lucknow, Bengaluru, Mumbai, Pune, Hyderabad and Haryana). These included 24 pieces of prose (in the form of a personal essay or 'blog'), nine poems, three pieces of visual art and one video. Four participants had two submissions each and these were treated as individual data files for analysis. The analysis established four themes: 1) "Living through difficulties"; 2) "Mental health in context"; 3) "Managing one's mental health"; and 4) "Breaking stigma and sharing hope". Corresponding sub-themes and illustrative quotes are presented below under each theme, along with frequency counts reflecting coverage of sub-themes across submissions.

Theme 1: Living through difficulties

This theme was comprised three sub-themes: "Powerful, painful thoughts and feelings"; "Loneliness and isolation"; and "Mental health as a daily struggle".

Powerful, painful thoughts and feelings (29/37)

Most narratives included descriptions of intense distress, pain, suffering and hopelessness. These often emphasized the palpable embodied nature of negative thoughts and feelings as being physically overwhelming and burdensome. Five narratives explicitly referred to suicidal thoughts and three spoke explicitly about self-harm.

"It was the first time when I resorted to self-inflicting physical pain as a coping mechanism. The idea was overwhelming and scary but the mental trauma was too much to handle. I couldn't think clear and grabbed my blade." (Male, 23)

A number of narratives described experiences of gradual onset of mental health problems, as well as sudden moments of realisation after reaching a "breaking point". Many narratives described feeling powerless and without control and described additional fear, guilt, shame and blame experienced towards themselves as a result of their mental health or "failure" to control thoughts and feelings. Many narratives spoke about a desperate desire to make sense of their experience, of asking "why" and what this meant for their identities.

For peer review only - http://bmjopen.bmj.com/site/about/guidelines.xhtml

"Anxiety and depression felt like parts of me that would take over at different times. Seeing yourself as mentally unwell makes you feel helpless about how to handle it." (Male, 23)

Loneliness and isolation (20/37)

Many narratives spoke about intense feelings of isolation and loneliness.

"I am alone on a never ending road to nowhere. I am enthralling the darkness, which blinds, even the light." (Female, 24)

Some participants described the tension between their desire for connection while simultaneously wishing to isolate themselves. Many talked about difficulties conveying their mental health experiences to others due to a lack of trust or fear of rejection if they opened up.

"I have no idea how to talk about depression with my peers, colleagues, relatives or friends. There are times when I isolate myself completely, avoiding any communication." (Male, 25)

Mental health as a daily struggle (15/37).

A sense of burden and day-to-day struggle with mental health problems was reflected in a number of the narratives. Some authors likened their experience to that of being in fight, war or battle while others referred to feeling trapped, imprisoned, shackled or in a dungeon. Narratives conveyed a strong sense of an ongoing effort and fatigue both in managing their mental health and keeping up with the tasks of daily life.

*"I feel caged from within. It's exhausting to keep up with the struggle.
I am afraid I might be losing the battle. A part of me is dying everyday."
(Male, 25)*

Many narratives spoke about putting on a mask or "faking" their way through daily life. They talked about how sleeping, watching TV, smoking, binge eating or drinking became ways to fill a "void" or achieve temporary relief, with some authors highlighting these were maladaptive coping methods which made them feel worse in the longer-term.

Theme 2: Mental health in context

This theme was comprised two sub-themes: "Personal, social and cultural context"; and "Knowledge, attitudes and stigma".

Personal, social and cultural context (14/37)

Some young people's narratives described the personal context of their mental health difficulties in terms of child abuse or growing up with a parent with mental health problems, while others alluded to wider societal factors and influences. Several authors otherwise mentioned focal precipitating events such as bereavement or being in an abusive relationship.

"Somewhere in myself I did know we wouldn't get married owing to conservative families and different religious faiths. We still hoped though and went ahead anyway. Although it gave us a space to ourselves, it gave him the access to beat me up, and no one else knew." (Female, 30)

Some narratives spoke about academic and career pressures and high expectations to "achieve" held by themselves, family members and other people around them. They spoke about how acknowledging one's own mental health needs was seen as personal weakness, particularly within a culture focused on "productivity" and achievement.

For peer review only - http://bmjopen.bmj.com/site/about/guidelines.xhtml

"I felt guilty about not working hard. I felt if I took a small break, disconnected myself or working a little less, admitting that I felt exhausted, I would be letting people down." (Female, 28)

Some authors wrote about their own socio-economic privilege, class, caste and education and how these factors have affected their ability to access support for their mental health problems.

"I have been fortunate enough to be able to access appropriate care for my mental health condition. I was lucky that I could take a break from work and give myself some time to get better. A majority of people struggling with mental health problems do not have this." (Female, 29)

Knowledge, attitudes and stigma (18/37)
Multiple narratives described a general lack of knowledge or understanding about mental health and experiences of negative and stigmatising attitudes. Several authors identified how others' preconceived ideas about "who has mental health", what it means to be a "man" or about their own identities resulted in denial or dismissal of their experiences.

"People told me, 'just cheer up', not to use depression as an 'excuse' and that 'medicine is not the answer'." (Female, 25)

A number of participants expressed negative attitudes, shame and guilt towards their own mental health or diagnosis and described struggling to come to terms with their difficulties. For some self-stigma or perceived public stigma had acted as a barrier to seeking help.

"In the beginning, I wasn't ready to accept the fact that there was something wrong, something that would make me look imperfect. I just wanted to be 'normal', like everyone else in my college." (Female, 25)

Several authors highlighted the need for accurate information about mental health so that people can identify when they have a problem and seek appropriate help.

"Awareness and access to information needs to increase. Then many more people can identify problems they might be facing and get the right kind of help they need and in time." (Female, 29)

Theme 3: Managing one's mental health
This was comprised three sub-themes: "Psychosocial support"; "Healthcare"; and "Self-care and recovery".

Psychosocial support (16/37)
Many narratives spoke about the key roles played by friends and family in providing emotional and practical support. Conversely, some narratives described a lack of support from their networks as being an additional burden. The importance of being able to talk with others about their mental health, and to be heard and not to be judged, was expressed strongly across young people's narratives.

"It did not really help when my peers made no effort to show compassion towards me or understand what I was going through." (Male, 25)

"I began to truly realize the importance of having family and friends around me, people who understood me. All I needed was someone to lend me an ear, give me the space to break down." (Male, 25)

Other authors described positive experiences of friends and family encouraging and enabling them to

For peer review only - http://bmjopen.bmj.com/site/about/guidelines.xhtml

access professional help. Awareness and knowledge about mental health by family and friends was seen as connected to their willingness and ability to offer meaningful support.

"It became my job to educate my family about my condition. They are nice people, they care about me but I could see through their helpless eyes, they were clueless about how to help me…" (Female, 29)

Healthcare (21/37)
Young people described varied experiences of mental healthcare, ranging from outpatient sessions with psychologists and/or psychiatrists to hospital admissions. Some narratives pointed to the effort and courage needed to access mental health services in the first place, and that finding the right diagnosis or treatment takes time. Several authors described professional help as having been pivotal to their recovery.

"Therapy taught me to notice my ways of thinking and responding to things. I learned ways to break unhelpful patterns and to build on the helpful ones." (Male, 28)

A number of narratives described positive experiences of prescribed psychotropic medication.

"When I started taking medication I felt calm for the first time in my life. It was like I had been stumbling around in the darkness and suddenly it was light. The voices I heard finally were quiet." (Female, 25)

Some participants who found medication effective nevertheless expressed a reluctance or dislike of taking medication and a desire not to do so in the future. Others described negative experiences of medication and of being medicated against their will.

"My voices didn't like the meds. While they made my life hard, at least I felt something. Being dragged to the psychiatrist felt like a punishment when I had done nothing wrong." (Female, 23)

Self-care and recovery (26/37)
Many narratives described experiences of recovery or improvements in mental health. Some participants shared experiences of self-care methods which helped them to maintain their well-being. These included taking care of their physical health; making changes to their relationship with work or study; and finding ways to express themselves through creative activities or talking to others.

"I have made changes in my office, making time in our meetings to talk about how we are feeling. It was a bit strange at first, but it soon became a vital part of how we worked together and supported each other. (Female, 28)"

Many narratives described a journey towards self-awareness and acceptance of themselves and their mental health difficulties as central to their recovery. Some authors conveyed the importance of holding on to hope through the difficult times and an acceptance that the future would contain ups and downs.

"I still have my bad days but I am better at dealing with them now. True happiness still feels like a distant dream and thoughts about my future worry me. But I know I shall get there someday." (Female, 22)

Some authors note progress in relationships and achievements in their education or careers accomplished in spite of mental health difficulties. Others spoke about personal growth and learning achieved directly as a result of the experiences of mental health problems. Across the narratives authors described their identity as not being limited by their mental health or illness.

For peer review only

For peer review only - http://bmjopen.bmj.com/site/about/guidelines.xhtml

"Experiencing a mental disorder taught me a lot about my own self. It made me grow and become stronger. It was the beginning of a new journey. I got back to what I loved doing, painting and writing!" (Female, 19)

Theme 4: Breaking stigma and sharing hope
The majority of narratives contained messages directed at readers with mental health problems as well as a broader audience. Within these messages two sub-themes were identified; "Desire to help others" and "Desire to break stigma".

Desire to help others (18/37)
Several authors shared the intention that their own story might help others by giving them hope, courage and reassurance that "you are not alone". Some authors spoke about positive experiences of sharing their mental health stories in their personal and professional lives and how helping others also helps them. Several authors gave specific advice to readers such as encouraging them to seek professional help. Across the narratives there was a strong desire to convey that life can get better and to encourage readers not to 'give up'.

'I hope I can help people to free themselves from being stuck in their own difficulties' (Female, 20)

Desire to break stigma (14/37)
Several authors identified their motivation for sharing their story in terms of normalising mental health problems and breaking stigma. Some narratives spoke about the importance of understanding mental health difficulties as a normal part of being human; others emphasised that mental health difficulties were "real" and equally important as problems affecting physical health.

"Mental illness is actually no different than a physical illness. It is just the same as having a cold, or a fracture or any physically manifested disease that you see a doctor for." (Female, 26)

The importance of mental health dialogue both personally and more broadly in society came out across many narratives Authors emphasised the courage required to disclose the state of ones' own mental health and stressed the powerful impact it can have on personal recovery and breaking wider stigma.

"We must talk about mental health at every level - in schools, at work, with medical professionals and in the media. We need more information and awareness so that people can spot problems and get help. It's not just OK to talk, it is absolutely essential!" (Female, 29)

DISCUSSION

This study set out to explore the lived experiences of mental health problems and recovery from a sample of young people in India, based on mixed-media submissions to a public website. Four overarching themes were identified of 1) Living through difficulties; 2) Mental health in context; 3) Managing one's mental health; and 4) Breaking stigma and sharing hope. Almost all submissions contained personal experiences of distress with the embodied experience of mental health difficulties as physically and mentally overwhelming and burdensome. Loneliness, isolation and a strong desire for connection were prominent, mirroring findings from qualitative studies of youth narratives obtained in high-income countries**[26,31]**.

The expression and sharing of such painful experiences appeared to be an important function of the It's Ok To Talk website. This is consistent with evidence that online spaces are commonly used by people with stigmatised health conditions for personal expression and social connection**[19-20]**. Informality and anonymity of the internet is emphasized in the extent to which it reduces the need to self-identify**[16]**.

For peer review only - http://bmjopen.bmj.com/site/about/guidelines.xhtml

Youth consulted during the design of the It's Ok To Talk website also felt that most submitters might not wish to disclose their identities. However, although the website offered anonymity, most participants (89% in this study) chose to include their name, age and location. This could be a reflection of the strong desire to be heard and recognised.

Many participants described their mental health difficulties within the context of broader socio-economic and cultural contexts. These submissions went beyond mere description of internal experiences to explore and challenge the societal and political contexts of psychological distress, offering a rich and multi-dimensional understanding of mental health. In particular, young people's narratives explained how rigid social norms can result in oppressive and unrealistic expectations, exacerbating stress and contributing to poor mental health. In India, academic failure and stress have been noted in verbal autopsies of young people and as a predictor of suicidal ideation**[32]**. This is also consistent with research in other developing countries which has found academic, interpersonal and family difficulties to be among the key social determinants of poor mental health and suicide in young people**[4, 33-34]**.

Participants raised concerns regarding equity of access to mental health care, and acknowledged structural barriers that prevented the majority of Indians from finding or otherwise accessing care. Consistent with extensive previous research on the pervasive effects of mental health stigma on help-seeking, both public and self-stigma also limited young people's access to services **[35-36]**. The influence of stigma was prominent in narratives around recovery, and particularly the finding that acceptance of mental health problems appeared to be a central part of many participants' journeys of recovery. For some young people, sharing their story on the website was part of this process. This is also consistent with literature on the impact of disclosure on self-stigma**[25]**. Research has shown that selective online disclosure of potentially stigmatizing health information may increase self-confidence, facilitate connections with similar others online and allow individuals to feel more accepting about their condition in offline settings**[6]**. In this study, in addition to fostering acceptance, many participants discussed other aspects of their lives and achievements, and voiced that their mental health problems alone did not define them as a person.

Participants expressed mixed experiences of mental healthcare. In contrast to the bloggers studied by Marcus et al (2012), the young people in this study were largely positive about their experiences of mental health professionals, and made recommendations to others about seeking professional help. Considering medication specifically, attitudes were mixed and predominantly negative. There is evidence of possible links between stigma and discontinuation of medication for mental health problems**[37-39].** It would be important for future qualitative research to explore possible influences of stigma, fear of side effects and individual values on attitudes towards medication.

Participants variously expressed personal motivations to mitigate public stigma, to reduce self-stigma and to help others. For instance, to reassure others they are not alone in their struggle and normalise mental health as part of the human experience. The need for better information and understanding of mental health was seen as central to this process. Relatedly, participants emphasised the importance of broadening the mental health discourse to encompass social spaces ranging from homes, educational institutions, workplaces and community settings. Similarly, other research has indicated that online communities can serve as powerful venues for people to challenge stigma through personal empowerment and by providing hope to others**[17]**.

Limitations

Limitations of this study included a convenience sample resulting in a narrow demographic cohort of self-selected, highly educated, English speakers (the website is in English) with access to the internet. Hence, participants are unlikely to be representative of the general youth population in India, which is a middle-income country with large disparities in internet access, especially among women and individuals from

For peer review only - http://bmjopen.bmj.com/site/about/guidelines.xhtml

lower socioeconomic status[40]. This is also consistent with US data showing that individuals who share illness experiences online appear to be more highly educated than the general population[41]. A further limitation is that young people's mental health problems were self-reported and could not be differentiated into diagnostic or other clinical categories. This study also did not include any standardised measures to understand impacts on authors or readers of the website and findings do not tell us about possible benefits or risks of online narratives for authors or readers. For example, some research has identified risks associated with obtaining advice from peers with unknown credentials or setting of unrealistic expectations due to learning about others' health experiences[42]. Further research is needed to explore these important questions.

Notwithstanding these limitations, the study adds to the growing literature on digital communication and personal narratives, and how these can play a role in addressing the needs of young people living with mental health problems. Further efforts are required to determine whether similar online spaces can be used successfully by young people from lower socioeconomic groups, with lower education levels, from rural areas, and from underrepresented or marginalized population subgroups. The launch of a Hindi version of the It's Ok To Talk website is planned for 2019, with the aim to extend the reach of the site to include a large number of of young internet users in India whose primary computing language is Hindi.

Conclusions

Social media offers opportunities to share personal narratives without temporal or spatial barriers[12]. Future research is needed to expand on the exploratory work described here and assess the effects of self-disclosure through online narratives. This is important both for promoting the mental health and wellbeing of those who have made these disclosures, and on attitudes regarding mental health problems in the wider community.

Such personal narratives highlight important gaps and needs in addressing young people's mental health needs. They also offer a window into young people's priorities in their own words and provide specific themes and nuanced key messages for awareness and help-seeking for future anti-stigma public engagement efforts in India or other similar settings.

"Listen, don't try to solve the problem."

"If you're struggling, know that it isn't your fault."

"You need to educate yourself so you can help others."

"There's no wrong time to get help."

"The most important thing to remember is that you're not alone."

For peer review only

CONTRIBUTORSHIP

PPG, ESH, DM, JN and VP designed the study with the support of SP and RS and PPG, ESH and RS coded the data, JN served as an auditor, and all authors contributed to interpretation of the data. PG and ESH wrote the manuscript with support from DM, SP, JN, RS and VP. All authors gave final approval of the version to be published.

COMPETING INTERESTS

The authors declare that the research was conducted in the absence of any commercial or financial relationships that could be construed as a potential conflict of interest.

ACKNOWLEDGEMENTS

The authors would like to thank all the participants who provided their valuable time to share their experiences and contribute to the coding and review of the manuscript. They are also grateful to Sunanda Jalote and Siddharth Parambi for their contributions.

FUNDING

This project has received funding from the Wellcome Trust, UK.

ETHICS APPROVAL

Sangath Institutional Review Board (IRB)

DATA SHARING

Additional information is available by emailing pattie.gonsalves@sangath.in

For peer review only

REFERENCES

1. United Nations Population Fund (UNFPA). The power of 1.8 billion: adolescents, youth and the transformation of the future. 2014;5 https://www.unfpa.org/sites/default/files/pub-pdf/EN-SWOP14-Report_FINAL-web.pdf (accessed 26 Sep 2018).

2. Patel V, Saxena S, Lund C et al. The Lancet Commission on global mental health and sustainable development. *The Lancet* Published Online First: 2018. doi:10.1016/s0140-6736(18)31612-x

3. Patel V, Saxena S, Frankish H, et al. Sustainable development and global mental health—a Lancet Commission. Lancet 2016;378(10024):1143-1145. doi: https://doi.org/10.1016/S0140-6736(16)00208-7

4. Patel V, Ramasundarahettige C, Vijayakumar L, et al. Suicide mortality in India: a nationally representative survey. Lancet 2012;379(9834):2343-2351. doi:10.1016/S0140-6736(12)60606-0.

5. Ziebland S, Wyke S. Health and Illness in a Connected World: How Might Sharing Experiences on the Internet Affect People's Health?. Milbank Quarterly 2012;90:219-249. doi:10.1111/j.1468-0009.2012.00662.x

6. Naslund J, Aschbrenner K, Marsch L et al. The future of mental health care: peer-to-peer support and social media. Epidemiology and Psychiatric Sciences 2016;25:113-122. doi:10.1017/s2045796015001067

7. Kemp S. Digital in 2017: Global Overview - We Are Social. We Are Social. 2018.https://wearesocial.com/special-reports/digital-in-2017-global-overview (accessed 26 Sep 2018).

8. Patel V, Flisher A, Hetrick S et al. Mental health of young people: a global public-health challenge. The Lancet 2007;369:1302-1313. doi:10.1016/s0140-6736(07)60368-7

9. United Nations Children's Fund (UNICEF). The State of the World's Children 2017: Children in a Digital World. UNICEF. 2017.https://www.unicef.org/publications/index_101992.html (accessed 26 Sep 2018).

10. Brody H. Stories of Sickness. New Haven, CT: Yale University Press. 1987.

11. Shah C. Social Media and Social Networking. *Social Information Seeking* 2017;:29-42. doi:10.1007/978-3-319-56756-3_3

12. Betton V, Borschmann R, Docherty M et al. The role of social media in reducing stigma and discrimination. British Journal of Psychiatry 2015;206:443-444. doi:10.1192/bjp.bp.114.152835

13. Kaplan A, Haenlein M. Users of the world, unite! The challenges and opportunities of Social Media. Business Horizons 2010;53:59-68. doi:10.1016/j.bushor.2009.09.003

For peer review only - http://bmjopen.bmj.com/site/about/guidelines.xhtml

14. Kranzler E, Bleakley A. Youth Social Media Use and Health Outcomes: #diggingdeeper. Journal of Adolescent Health 2019;64:141-142. doi:10.1016/j.jadohealth.2018.11.002

15. Pantic I. Online Social Networking and Mental Health. Cyberpsychology, Behavior, and Social Networking 2014;17:652-657. doi:10.1089/cyber.2014.0070

16. Berger M, Wagner T, Baker L. Internet use and stigmatized illness. Social Science & Medicine 2005;61:1821-1827. doi:10.1016/j.socscimed.2005.03.025

17. Lawlor A, Kirakowski J. Online support groups for mental health: A space for challenging self-stigma or a means of social avoidance?. Computers in Human Behavior 2014;32:152-161. doi:10.1016/j.chb.2013.11.015

18. Burns J, Durkin L, Nicholas J. Mental Health of Young People in the United States: What Role Can the Internet Play in Reducing Stigma and Promoting Help Seeking?. Journal of Adolescent Health 2009;45:95-97. doi:10.1016/j.jadohealth.2008.12.006

19. Harvey K, Brown B, Crawford P et al. 'Am I normal?' Teenagers, sexual health and the internet. Social Science & Medicine 2007;65:771-781. doi:10.1016/j.socscimed.2007.04.005

20. Naslund J, Grande S, Aschbrenner K et al. Naturally Occurring Peer Support through Social Media: The Experiences of Individuals with Severe Mental Illness Using YouTube. PLoS ONE 2014;9:e110171. doi:10.1371/journal.pone.0110171

21. Rickwood D, Deane F, Wilson C. When and how do young people seek professional help for mental health problems? 2007;Med J Aust 2007;187:S35-39.

22. Itsoktotalk.in. (2018). It's Ok To Talk. [online] Available at: http://itsoktotalk.in/ [Accessed 26 Sep. 2018].

23. World Health Organization. (2016). World Health Day 2017. [online] Available at: http://www.who.int/campaigns/world-health-day/2017/campaign-essentials/en/ [Accessed 26 Sep. 2018].

24. Corrigan P, Kosyluk K, Rüsch N. Reducing Self-Stigma by Coming Out Proud. American Journal of Public Health 2013;103:794-800. doi:10.2105/ajph.2012.301037

25. Corrigan P, Morris S, Larson J et al. Self-stigma and coming out about one's mental illness. Journal of Community Psychology 2010;38:259-275. doi:10.1002/jcop.20363

26. Marcus M, Westra H, Eastwood J et al. What Are Young Adults Saying About Mental Health? An Analysis of Internet Blogs. Journal of Medical Internet Research 2012;14:e17.

For peer review only - http://bmjopen.bmj.com/site/about/guidelines.xhtml

doi:10.2196/jmir.1868

27. Ziebland S, McPherson A. Making sense of qualitative data analysis: an introduction with illustrations from DIPEx (personal experiences of health and illness). Medical Education 2006;40:405-414. doi:10.1111/j.1365-2929.2006.02467.x

28. Brownlow C, O'Dell L. Ethical Issues for Qualitative Research in On-line Communities. Disability & Society 2002;17:685-694. doi:10.1080/0968759022000010452

29. Eysenbach G, Wyatt J. Using the Internet for Surveys and Health Research. Journal of Medical Internet Research 2002;4:e13. doi:10.2196/jmir.4.2.e13

30. Bradley E, Curry L, Devers K. Qualitative Data Analysis for Health Services Research: Developing Taxonomy, Themes, and Theory. Health Services Research 2007;42:1758-1772. doi:10.1111/j.1475-6773.2006.00684.x

31. Midgley N, Parkinson S, Holmes J et al. Beyond a diagnosis: The experience of depression among clinically-referred adolescents. *Journal of Adolescence* 2015;44:269-279. doi:10.1016/j.adolescence.2015.08.007

32. Aaron R, Joseph A, Abraham S et al. Suicides in young people in rural southern India. The Lancet 2004;363:1117-1118. doi:10.1016/s0140-6736(04)15896-0x

33. Parikh R, Sapru M, Krishna M et al. 'It is lot like mind attack': A qualitative study of stress and coping among adolescents in India. *BMC Psychiatry* 2018. (In submission)

34. Patel V, Araya R, Chatterjee S. et al. Treatment and prevention of mental disorders in low-income and middle-income countries. The Lancet 2007;370:991-1005. doi:10.1016/s0140-6736(07)61240-9

35. Gulliver A, Griffiths K, Christensen H. Perceived barriers and facilitators to mental health help-seeking in young people: a systematic review. BMC Psychiatry 2010;10. doi:10.1186/1471-244x-10-113

36. Clement S, Schauman O, Graham T et al. What is the impact of mental health-related stigma on help-seeking? A systematic review of quantitative and qualitative studies. Psychological Medicine 2014;45:11-27. doi:10.1017/s0033291714000129

37. Sirey J, Bruce M, Alexopoulos G et al. Perceived Stigma as a Predictor of Treatment Discontinuation in Young and Older Outpatients With Depression. American Journal of Psychiatry 2001;158:479-481. doi:10.1176/appi.ajp.158.3.479

For peer review only - http://bmjopen.bmj.com/site/about/guidelines.xhtml

38. Corrigan P. How stigma interferes with mental health care. *American Psychologist* 2004;59:614-625. doi:10.1037/0003-066x.59.7.614

39. Livingston J, Boyd J. Correlates and consequences of internalized stigma for people living with mental illness: A systematic review and meta-analysis. Social Science & Medicine 2010;71:2150-2161. doi:10.1016/j.socscimed.2010.09.030

40. Internet access grows worldwide but remains higher in advanced economies. Pew Research Center's Global Attitudes Project. 2018. http://www.pewglobal.org/2016/02/22/internet-access-growing-worldwide-but-remains-higher-in-advanced-economies/ (Accessed 11 Oct 2018).

41. Naslund J, Aschbrenner K, Araya R et al. Digital technology for treating and preventing mental disorders in low-income and middle-income countries: a narrative review of the literature. *The Lancet Psychiatry* 2017;4:486-500. doi:10.1016/s2215-0366(17)30096-2/

42. Naslund J, Aschbrenner K, McHugo G et al. Exploring opportunities to support mental health care using social media: A survey of social media users with mental illness. *Early Intervention in Psychiatry* Published Online First: 2017. doi:10.1111/eip.12496

For peer review only

Consolidated criteria for reporting qualitative studies (COREQ): 32-item checklist

No	Item	Description	Location in the manuscript (Section, pg no.)
**Domain 1: Research team and reflexivity**			
Personal Characteristics			
1.	Interviewer/facilitator	NA	NA
2.	Credentials	PPG: MSc<br>ESH: DClinPsy<br>RS: MSc<br>JN: PhD	Title page,1
3.	Occupation	PPG, ESH and RS are presently employed with Sangath, a mental health research NGO.<br>JN is presently a Post-Doc candidate at Harvard Medical School.	Title page,1
4.	Gender	PPG, ESH, RS: Female<br>JN: Male	NA
5.	Experience and training	PPG and RS had acquired training on qualitative research in MSc degree programs and have more than 3 years of research experience including qualitative research.<br>ESH is a clinical psychologist with training in qualitative research design and practice. She has previously conducted and published qualitative research.<br>JN has extensive experience with qualitative analysis.	Title page, Methods, 5-6
Relationship with participants			
6.	Relationship established	Yes	Methods, 6
7.	Participant knowledge of the interviewer	Study participants and FGD participants were briefed about the purpose of the study and written	Methods, 6

For peer review only

		information was also provided by email and telephone. The necessary ethical approvals were received before commencement of study.	
8.	Interviewer characteristics	There are no potential sources of bias.	NA
**Domain 2: study design**			
Theoretical framework			
9.	Methodological orientation and Theory	We followed an integrated inductive-deductive approach to thematic content analysis	Methods,
Participant selection			
10.	Sampling	The study's sampling frame included submissions made to a web platform during the first year of its operation. Participants involved in a small FGD and paraphrasing activities were randomly selected from among the list of submissions originating from New Delhi where the researchers were based.	Methods, 5-6
11.	Method of approach	All prospective participants to the analysis and the FGD were approached by email.	Methods, 6
12.	Sample size	33	Methods, 5
13.	Non-participation	11	Methods, 5
Setting			
14.	Setting of data collection	The study's sampling frame included submissions made to a web platform during the first year of its operation.<br>The small participant FGD was conducted at the office premises of the researchers.	Methods, 8

For peer review only

15.	Presence of non-participants	No	
16.	Description of sample	The study's sampling frame included submissions made to the It's OK to Talk web platform during the first year of its operation (April 2017 - March 2018). Eligible submissions were English-language first-person accounts of self-identified mental health problems, submitted in any media format for online publication by authors aged 18-31 years and of Indian nationality. 37 submissions by 33 authors aged 19-31 years (mean age 22 years) from 7 Indian cities (New Delhi, Lucknow, Bengaluru, Mumbai, Pune, Hyderabad and Haryana) were included for analysis.	Methods, 5
Data collection			
17.	Interview guide	The data included in the analysis was guided only by the information on the website to share stories of mental health and raise awareness about youth mental health problems.<br><br>Yes, the interview guide for the FGD was prepared by the authors for the purpose of this study.	Introduction, 4<br>Methods, 8
18.	Repeat interviews	No	-
19.	Audio/visual recording	No	-
20.	Field notes	Field notes were made during the FGD	Methods, 6
21.	Duration	The FGD lasted 90 minutes	Methods, 6
22.	Data saturation	NA	-
23.	Transcripts returned	NA	-
**Domain 3: analysis and**			

For peer review only

**findings**			
Data analysis			
24.	Number of data coders	3 coders, 1 auditor	Methods, 5-6
25.	Description of the coding tree	Deductive and inductive codes were developed. Related codes were ordered into meaningful categories to convey interrelated ideas.	Methods, 5-6
26.	Derivation of themes	Yes	Methods, 5-6
27.	Software	nVivo 10	Methods, 6
28.	Participant checking	Focus group participants provided quality assurance and feedback on the analysis	Methods, 6
Reporting			
29.	Quotations presented	All personally identifiable data was removed and all direct quotations were paraphrased to ensure anonymity.	Results, 6-11
30.	Data and findings consistent	Yes	Results, 6-11
31.	Clarity of major themes	Yes	Results, 6-11
32.	Clarity of minor themes	Yes	Results, 6-11

For peer review only
